# Spatiotemporal Dynamics, Climate Lag Effects, and Environmental Associations of Vegetation Cover Across the Western Sichuan Plateau During 2001–2024

**DOI:** 10.3390/plants15193040

**Published:** 2026-10-04

**Authors:** Yu Feng, Peng Ye, Xiyao Hua, Jialong Zhong, Yong Yao, Zhiming Gong

**Affiliations:** 1 School of Data Science, Chengdu Technological University, Chengdu 611730, China; fengyu@cdtu.edu.cn; 2 Faculty of Geosciences and Engineering, Southwest Jiaotong University, Chengdu 611756, China; yep@swjtu.edu.cn; 3 School of Management Science, Sichuan University of Science & Engineering, Yibin 644000, China; jialogn@gmail.com; 4 The 7th Geological Brigade of Sichuan, Leshan 614099, China; 5 School of Materials and Environmental Engineering, Chengdu Technological University, Chengdu 611730, China

**Keywords:** Western Sichuan Plateau, fractional vegetation cover, climate lag effect, XGBoost-SHAP, spatial environmental associations

## Abstract

Vegetation dynamics in alpine ecosystems are highly sensitive to climate and environmental changes. However, the spatial heterogeneity of vegetation cover and its lagged associations with climate variability remain insufficiently understood. This study estimated fractional vegetation cover (FVC) based on MOD13Q1 NDVI data. The Sen’s slope and Mann–Kendall tests were employed to describe trends in FVC change, while pixel-based Pearson correlation analysis was used to quantify lagged associations (0–12 months) between temperature and precipitation and FVC. Furthermore, the XGBoost–SHAP framework was used to quantify nonlinear environmental associations with the spatial variation in FVC. The results showed that, from 2001 to 2024, the growing-season FVC on the Western Sichuan Plateau exhibited a significant increasing trend, with an average value of 0.7062 and a rate of 0.0018 yr^−1^ (R^2^ = 0.5040, *p* < 0.001). Areas with high and relatively high FVC values accounted for 75.7873% of the study area, indicating generally favourable vegetation conditions with substantial spatial heterogeneity. Both temperature and precipitation exhibited lagged associations with FVC, with long-term lags (7–12 months) accounting for 45.6600% and 45.0768% of the study area, respectively. After integrating topography and land-cover information, the explanatory power of the XGBoost model was enhanced, with the R^2^ value increasing from 0.2181 in the climate-only model to 0.6393 in the full ecological model (RMSE = 0.1800, MAE = 0.1263). SHAP analysis indicated that DEM (elevation), LC_10 (grasslands), and TMEAN (Temperature_2m) made the largest contributions to the modelled spatial variation in FVC, accounting for 39.0853%, 17.3644%, and 10.3859%, respectively. These results indicate persistent associations between FVC spatial heterogeneity and topographic, land-cover, and climatic gradients.

## 1. Introduction

Vegetation dynamics are a key indicator of terrestrial ecosystem responses to global environmental change and play an important role in regulating carbon cycling, energy exchange, and ecosystem stability [1,2,3]. Within the persistently warming climate, changes in vegetation of alpine ecosystems are attracting increasing attention, as these regions are characterised by strong climatic limitations, a short growing season and high sensitivity to environmental fluctuations [4,5]. To understand how alpine vegetation responds to changing climatic conditions, it is essential to predict trends in ecosystem evolution and to develop effective conservation strategies under future climate scenarios.

The Tibetan Plateau, known as the “Third Pole” of the Earth, is recognised as one of the largest alpine ecosystems in the world. It plays a crucial role in regional climate regulation, water resource conservation and biodiversity maintenance [6,7,8,9]. The eastern margin of the Tibetan Plateau, including the Western Sichuan Plateau, is characterised by complex topography, marked elevation gradients, and diverse vegetation types ranging from alpine meadow and alpine grassland to shrub and forest ecosystems [10,11,12]. These environmental gradients result in significant spatial heterogeneity in vegetation distribution and ecological processes [13,14]. However, alpine ecosystems in this region are highly vulnerable to climate warming and changes in hydrothermal conditions, due to their limited thermal availability and fragile ecological structure [15]. Therefore, quantifying vegetation changes and identifying their environmental drivers is essential to understanding ecosystem responses in high-altitude regions.

Vegetation indices derived from remote sensing technology provide an effective method for monitoring vegetation dynamics across large spatial and temporal scales [2,16,17]. Among these indices, the normalised difference vegetation index (NDVI) has been widely used to assess vegetation activity and ecosystem changes [6,18]. The fractional vegetation coverage (FVC) estimated from NDVI provides a more direct reflection of vegetation structural characteristics and has been extensively applied in ecological assessments [19,20]. Previous studies have used linear regression, trend analysis and change detection methods to investigate vegetation trends on the Tibetan Plateau [7,11,21]. However, most studies have focused on identifying patterns of vegetation greening or browning, and the mechanisms driving vegetation responses to environmental changes remain insufficiently understood.

The climate–vegetation interactions in alpine ecosystems are often more complex than instantaneous linear responses [22,23,24]. The growth of vegetation may be influenced by historical climate conditions, which are linked to lagged climate–vegetation associations associated with soil moisture storage, thermal accumulation and physiological adaptation processes [14,25]. Temperature may regulate vegetation development through the cumulative supply of thermal energy, particularly in cold-limited alpine environments [26]. Meanwhile, the impact of precipitation may depend on delayed water infiltration, soil moisture persistence and vegetation hydration processes [14]. Although previous studies have examined climate–vegetation correlations, relatively few have quantified spatially explicit lagged responses between vegetation dynamics and multiple climatic factors across alpine regions [27]. Therefore, determining the optimal lag period and comparing the relative importance of temperature and precipitation are crucial for improving the understanding of climate–vegetation feedback mechanisms.

Furthermore, vegetation dynamics are influenced not only by climate but are also regulated by a combination of various interacting environmental factors [28,29]. Topographical conditions, ecosystem types and climatic variables collaborate to shape vegetation distribution and productivity, particularly in mountainous regions characterised by significant environmental gradients [11,20]. Traditional statistical methods usually assume linear relationships and may fail to capture the nonlinear interactions and threshold effects between environmental variables effectively [30]. Recent advances in the field of machine-learning provide new opportunities for exploring complex ecological relationships. Among these approaches, extreme gradient boosting (XGBoost) has demonstrated strong predictive performance in ecological modelling by capturing nonlinear relationships and interactions among predictors [31]. However, the limited explainability of machine learning models remains a challenge for ecological applications. The SHapley Additive exPlanations (SHAP) framework provides an effective solution by quantifying the contribution of individual variables to model predictions and revealing the ecological mechanisms underlying complex model outputs [32].

Although significant progress has been made in vegetation monitoring and the assessment of climate impacts, the long-term spatiotemporal characteristics of vegetation cover on the Western Sichuan Plateau still require further clarification. There remains a shortage of quantitative research into the lagged associations between temperature and precipitation and alpine vegetation growth. The relative contributions of climate, topography, and land-cover characteristics to the spatial variation in FVC, as well as their consistency across different periods, also remain insufficiently understood. Therefore, this study integrates remote sensing and climate data, trend analysis, lag analysis, and the XGBoost–SHAP-interpretable machine-learning framework to characterise FVC dynamics and environmental associations on the Western Sichuan Plateau between 2001 and 2024. The objectives of this study are (1) to describe the spatial distribution and temporal trends of FVC on the Western Sichuan Plateau; (2) to quantify lagged climate–vegetation associations between temperature and precipitation and FVC, and to identify the primary climate–vegetation response patterns; and (3) to quantify the nonlinear contributions of climatic, topographic, and land-cover variables to the spatial variation in FVC and examine their consistency across different periods using the XGBoost–SHAP framework. This study provides an integrated assessment of FVC spatial patterns, temporal trends, lagged climate–vegetation associations, and nonlinear environmental associations with FVC variability in a high-altitude region.

## 2. Results

### 2.1. Spatial Distribution and Temporal Dynamics of Vegetation Coverage

#### 2.1.1. Spatial Pattern of Multi-Year Mean FVC

**Figure 1:**
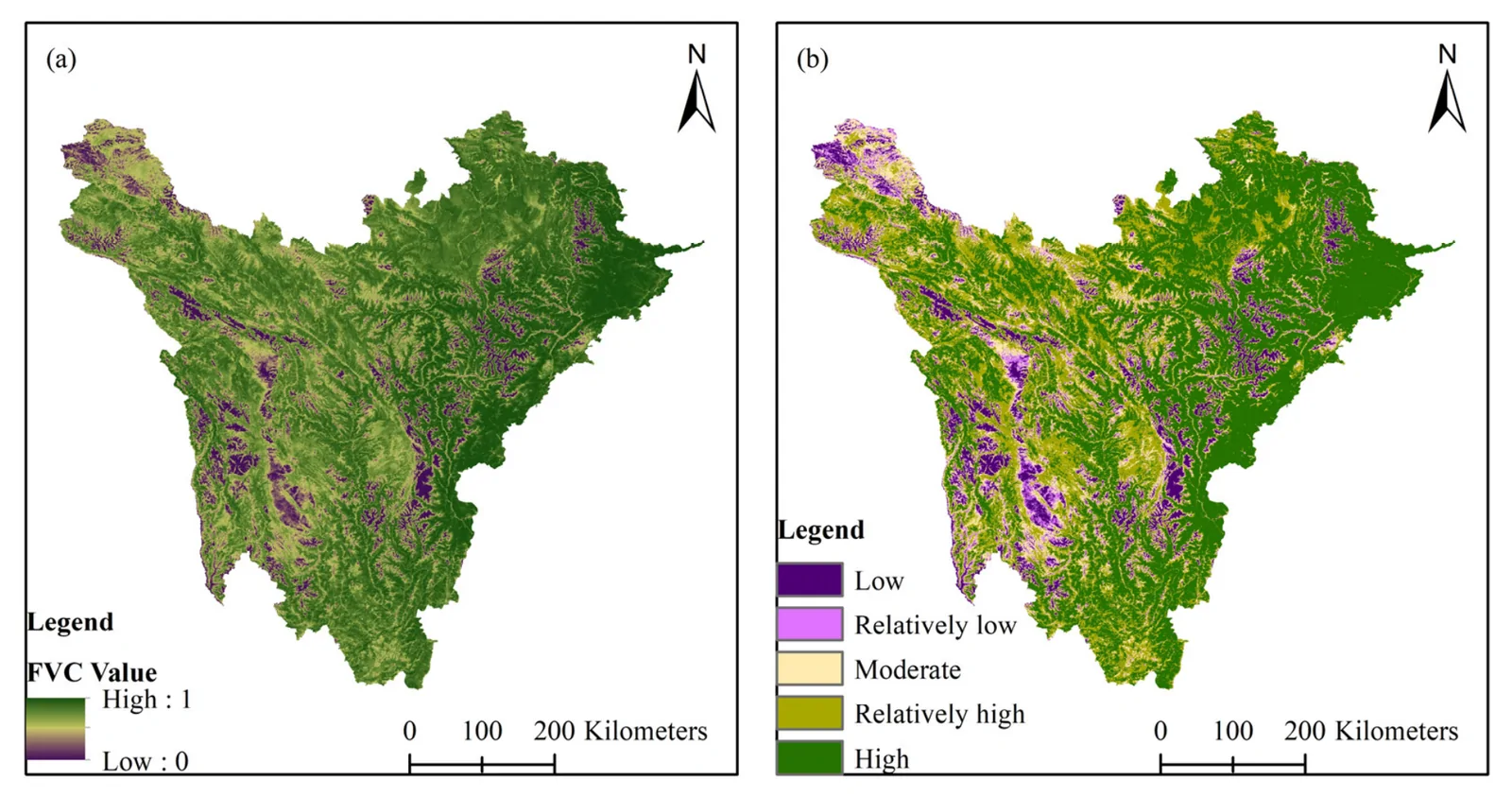
Spatial distribution of multi-year mean growing-season FVC during 2001–2024. (**a**) FVC value; (**b**) FVC level.

During the period 2001–2024, the multi-year mean growing season FVC values for the Western Sichuan Plateau exhibited spatial heterogeneity (Figure 1a). The regional vegetation pattern generally followed a distinct southeast–northwest gradient, characterised by higher FVC in the eastern and southeastern regions, and lower FVC in the western and southwestern regions. High FVC values were mainly found in the eastern mountainous areas and southeastern valleys, where relatively favourable hydrothermal conditions and diverse vegetation types, including forests and shrub ecosystems, support higher vegetation productivity. In contrast, low FVC values were primarily distributed in the western and southwestern high-elevation regions, where alpine climate constraints, low temperatures, short growing seasons, and limited water availability restrict vegetation development. The central region exhibited intermediate FVC values and represented a transition zone between densely vegetated mountainous ecosystems and sparsely vegetated alpine environments. This spatial pattern indicates that vegetation coverage across the Western Sichuan Plateau is strongly associated with large-scale environmental gradients, particularly elevation-related thermal constraints and ecosystem distribution patterns.

Based on FVC values, vegetation coverage was divided into five levels: low coverage (0–0.2), relatively low coverage (0.2–0.4), moderate coverage (0.4–0.6), relatively high coverage (0.6–0.8), and high coverage (0.8–1.0) (Figure 1b). Area statistics show that vegetation cover in the Western Sichuan Plateau was dominated by relatively high and high FVC categories (Table 1). High-coverage areas accounted for 46.9025% of the study region, while relatively high-coverage areas accounted for 28.8848%. Together, these two categories represented 75.7873% of the total area, indicating that most regions maintained relatively dense vegetation cover during the study period. Moderate-coverage areas accounted for 11.3830%, whereas relatively low and low-coverage areas accounted for 5.2387% and 7.5909%, respectively. These low-coverage regions were mainly associated with high-elevation alpine environments, where vegetation growth is strongly constrained by climatic limitations.

#### 2.1.2. Temporal Variation in Annual FVC

**Figure 2:**
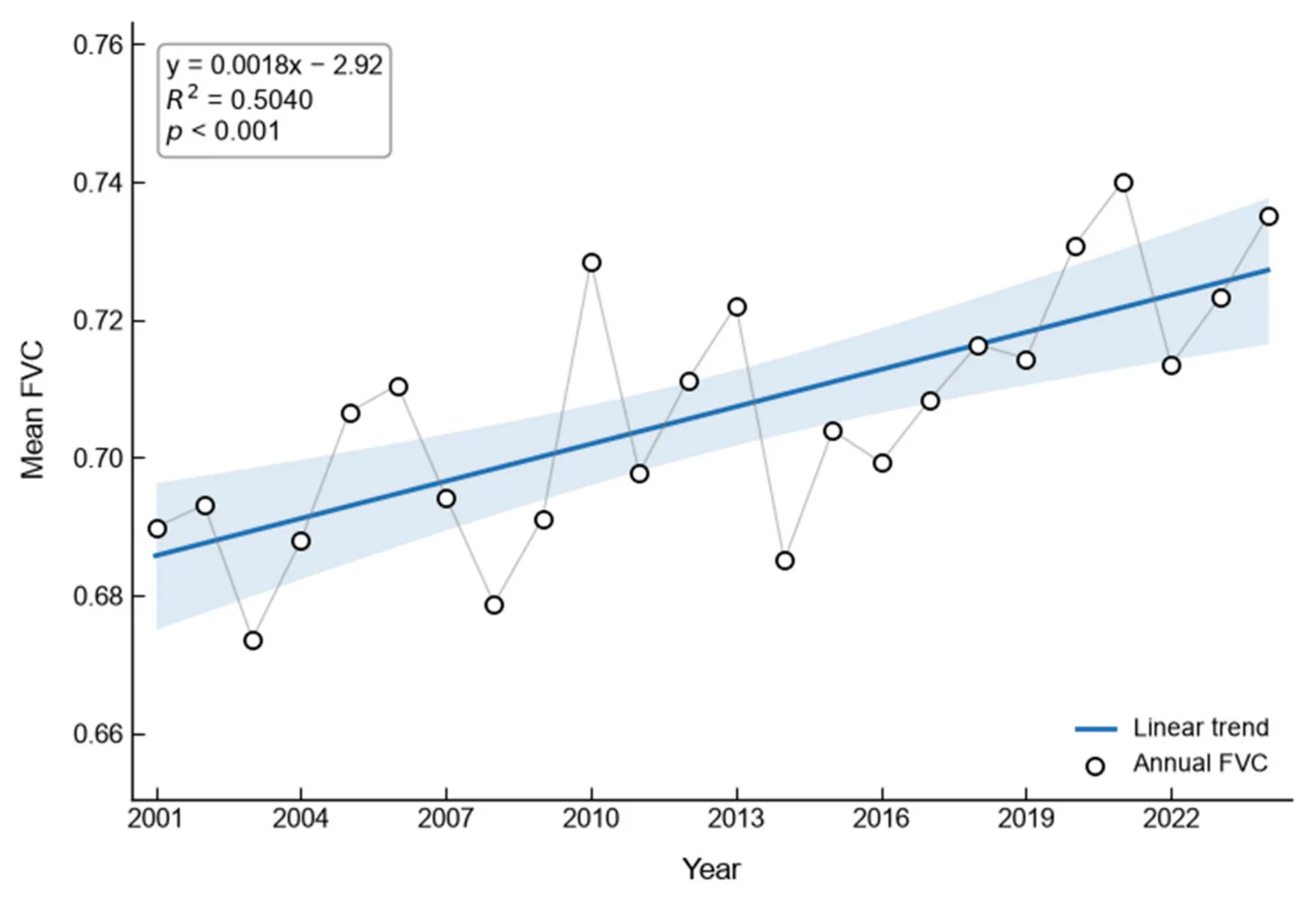
Temporal variation in mean growing-season FVC in the Western Sichuan Plateau during 2001–2024.

The annual growing-season mean FVC showed considerable inter-annual fluctuations but exhibited an overall increasing trend during 2001–2024 (Figure 2). The regional mean FVC ranged from 0.6737 to 0.7401, with an average of 0.7062 over the entire study period. The long-term trend analysis indicated that annual FVC increased significantly at a rate of 0.0018 yr^−1^ with an *R*^2^ value of 0.5040, and this trend was statistically significant (*p* < 0.001). This result suggests that, over the past 24 years, vegetation cover on the Western Sichuan Plateau has shown a sustained greening trend.

Although the long-term trend was significant, annual FVC also exhibited marked inter-annual fluctuations. The lowest value occurred in 2003 (0.6737), followed by a generally increasing but fluctuating pattern, with relatively low values in 2008 (0.6788), and 2014 (0.6851). In contrast, relatively high values were observed in 2021 (0.7401), 2013 (0.7221), 2010 (0.7284), and 2006 (0.7105). Despite these inter-annual fluctuations, FVC in 2024 reached 0.7351, which was considerably higher than the values in the early years of the study period. Overall, vegetation cover on the Western Sichuan Plateau showed a fluctuating but clearly increasing trend during 2001–2024, indicating a sustained greening of the alpine vegetation system.

#### 2.1.3. Spatial Patterns of Vegetation Change Trends

**Figure 3:**
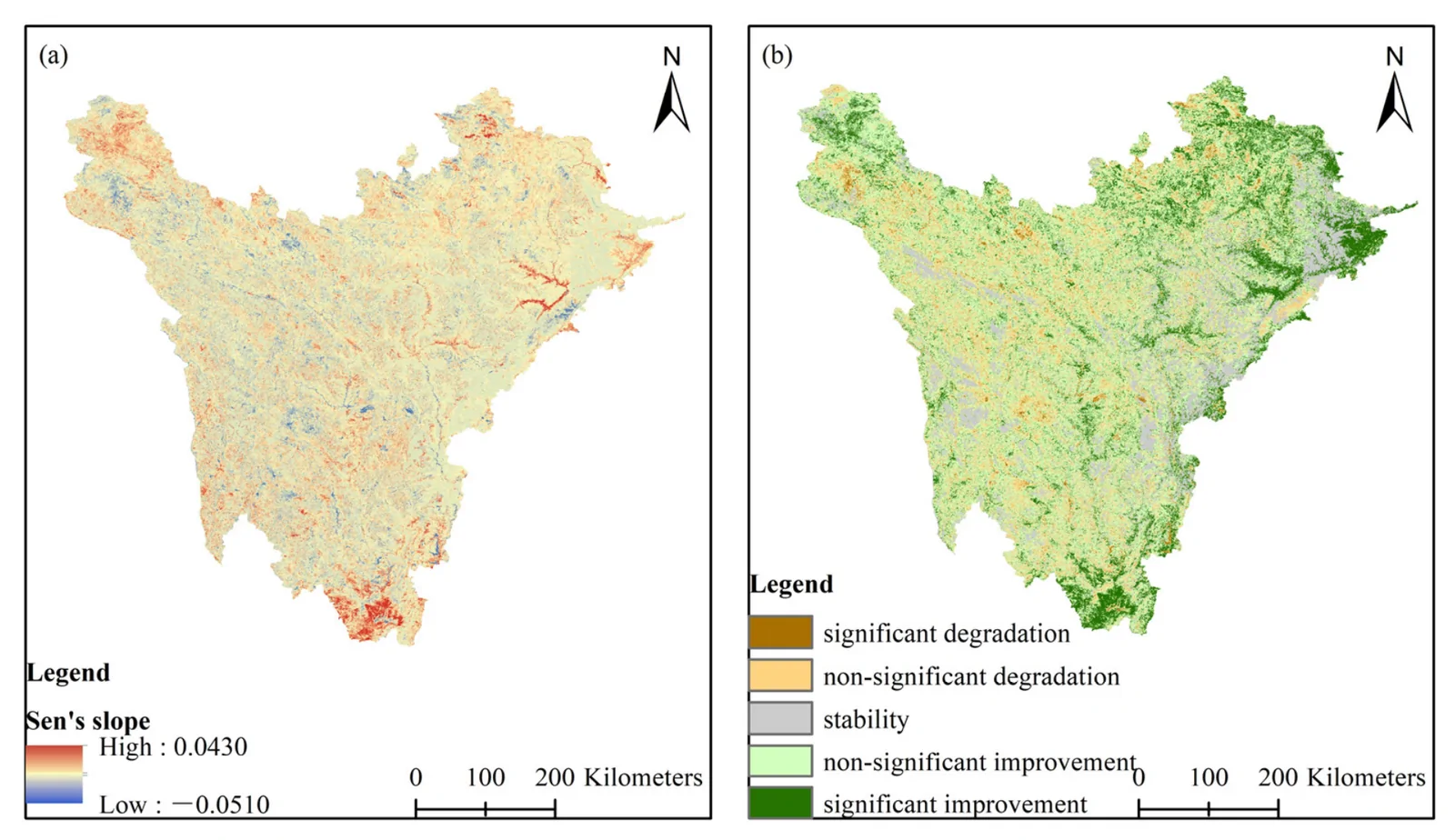
Spatial patterns of FVC trends during 2001–2024. (**a**) Sen’s slope values; (**b**) FVC change trends.

The spatial distribution of FVC trends in the Western Sichuan Plateau region also exhibited heterogeneity during 2001–2024 (Figure 3). Sen’s slope values ranged from −0.0507 to 0.0384 yr^−1^, indicating a relatively low rate of vegetation change at the regional scale. Positive and negative trends showed distinct spatial differentiation (Figure 3a). Positive trends (Sen’s slope > 0) were mainly distributed in the eastern, southeastern, and some mountainous regions, whereas negative trends (Sen’s slope < 0) were concentrated in western, southwestern, and locally central regions. The spatial distribution of these increasing and decreasing trends exhibited a mosaic pattern, reflecting the complex interactions between environmental gradients and ecosystem characteristics.

Based on Sen’s slope and the Mann–Kendall significance test, vegetation changes were classified into five categories: significant improvement, non-significant improvement, stability, non-significant degradation, and significant degradation (Figure 3b; Table 2). Among these categories, non-significant improvement was the dominant trend type, accounting for 47.0582% of the study area (154,486.0000 km^2^). These areas were widely distributed in the eastern, southeastern, and parts of the central plateau, indicating that vegetation recovery occurred over extensive regions, although the increasing trends were not statistically significant. Significant improvement accounted for 16.5638% (54,376.6250 km^2^) of the study area and was mainly distributed in eastern and southeastern regions. The combined area of significant and non-significant improvement reached 63.6220%, suggesting that vegetation greening was the predominant pattern across the Western Sichuan Plateau during 2001–2024.

In contrast, FVC degradation occurred in a smaller proportion of the study area. Non-significant degradation accounted for 21.5026% (70,590.3125 km^2^) and was mainly distributed in western and high-elevation regions. These areas exhibited declining FVC trends, but most changes did not reach statistical significance, suggesting relatively weak degradation signals. Significant degradation occupied only 1.9783% (6494.4375 km^2^) of the study area and was mainly located in localised high-elevation and ecologically sensitive regions. Stable areas accounted for 12.8971% (42,339.4375 km^2^) of the study area, primarily distributed in regions with limited vegetation variability, including lakes, rivers, snow-covered areas, and other low-vegetation-cover surfaces.

Overall, FVC improvement (significant and non-significant improvement) accounted for 63.6220% of the study area, whereas vegetation degradation (significant and non-significant degradation) accounted for 23.4809%. These results indicate that FVC generally exhibited an increasing trend across the Western Sichuan Plateau during 2001–2024, while degradation was mainly restricted to western and high-altitude regions.

### 2.2. Lagged Associations Between Vegetation and Climate Variability

#### 2.2.1. Lagged Associations Between Vegetation and Temperature Variability

**Figure 4:**
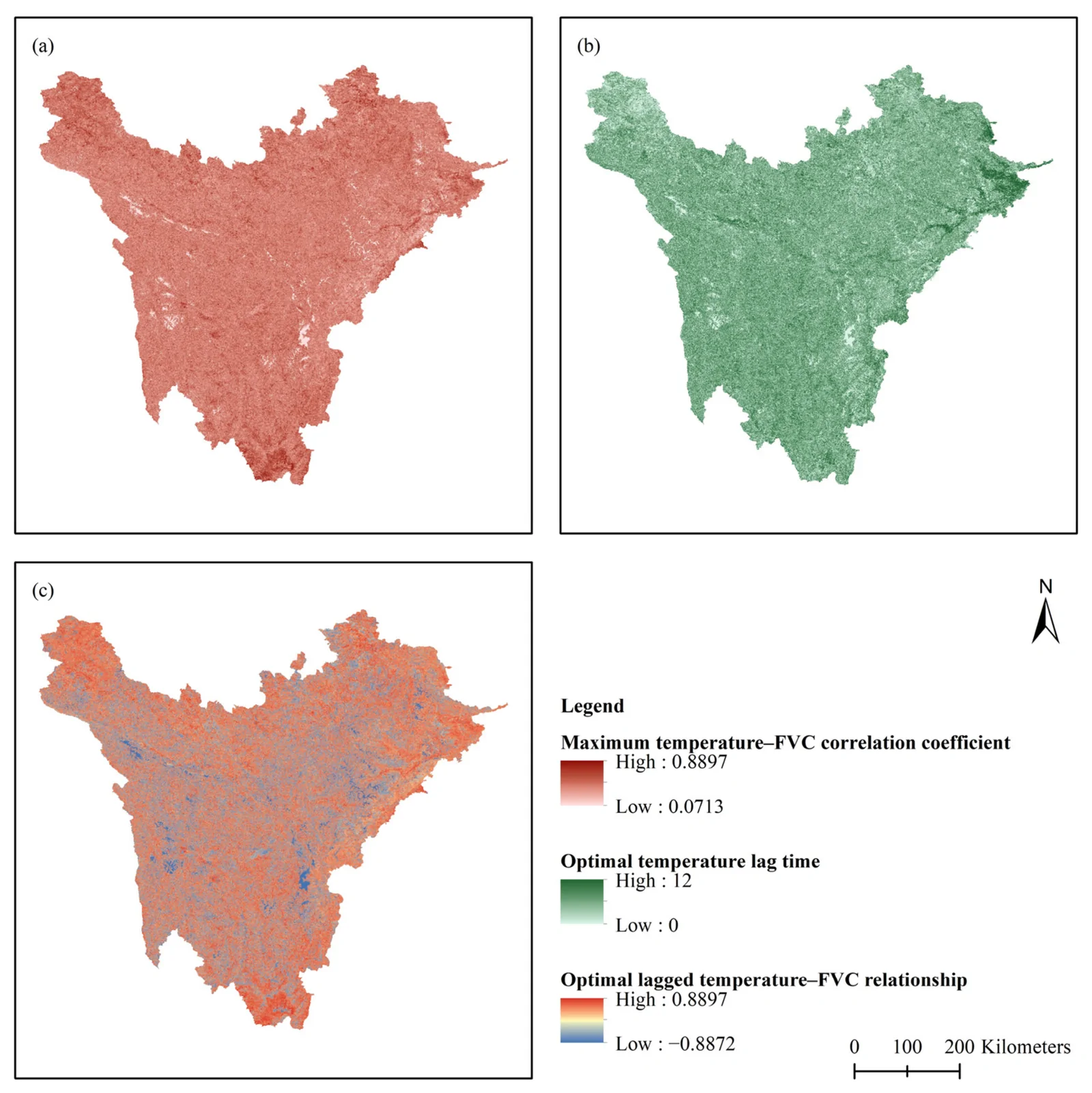
Lagged associations between temperature variability and vegetation growth across the Western Sichuan Plateau. (**a**) Maximum temperature–FVC correlation coefficient; (**b**) optimal temperature lag time; (**c**) optimal lagged temperature–FVC relationship.

The relationship between temperature variability and vegetation growth on the Western Sichuan Plateau exhibits spatial heterogeneity (Figure 4). Under different lag periods, temperature–FVC correlations varied noticeably, indicating that vegetation responses to thermal conditions were strongly dependent on temporal scales.

The maximum absolute correlation coefficient (|r|) between temperature and FVC ranged from 0.0713 to 0.8897, with high-correlation regions mainly distributed in high-elevation alpine ecosystems (Figure 4a). These regions are characterised by low temperatures and short growing seasons, in which temperature availability is a key environmental limiting factor for vegetation development. Changes in thermal conditions may significantly influence vegetation physiological processes, including growth initiation, leaf expansion, and photosynthetic activity. Compared with relatively warmer low-elevation regions, alpine zones exhibited stronger temperature sensitivity, suggesting that thermal limitation plays a more dominant role in vegetation conditions under cold environmental conditions.

The spatial distribution of optimal temperature lag time demonstrated clear regional differences (Figure 4b). The optimal lag period ranged from 0 to 12 months, indicating that temperature variability was statistically associated with FVC at different temporal offsets. Among different lag categories, long-term lag (7–12 months) occupied the largest proportion of the study area, followed by short-term lag (1–3 months), medium-term lag (4–6 months), and immediate response (0 months). Under the optimal lag conditions, temperature–FVC correlation coefficients ranged from −0.8872 to 0.8897 (Figure 4c). Positive correlations dominated most regions, indicating that vegetation growth was generally associated with favourable thermal conditions. However, negative temperature–FVC relationships occurred in some areas, suggesting that warming may not always coincide with higher FVC. The prevalence of long-term temperature lags indicates that antecedent temperature conditions were frequently associated with subsequent FVC, while the underlying mechanisms cannot be established from correlation analysis alone. Such lagged associations may be related to cumulative thermal conditions in alpine ecosystems, but this interpretation requires further physiological or process-based evidence.

#### 2.2.2. Lagged Associations Between Vegetation and Precipitation Variability

**Figure 5:**
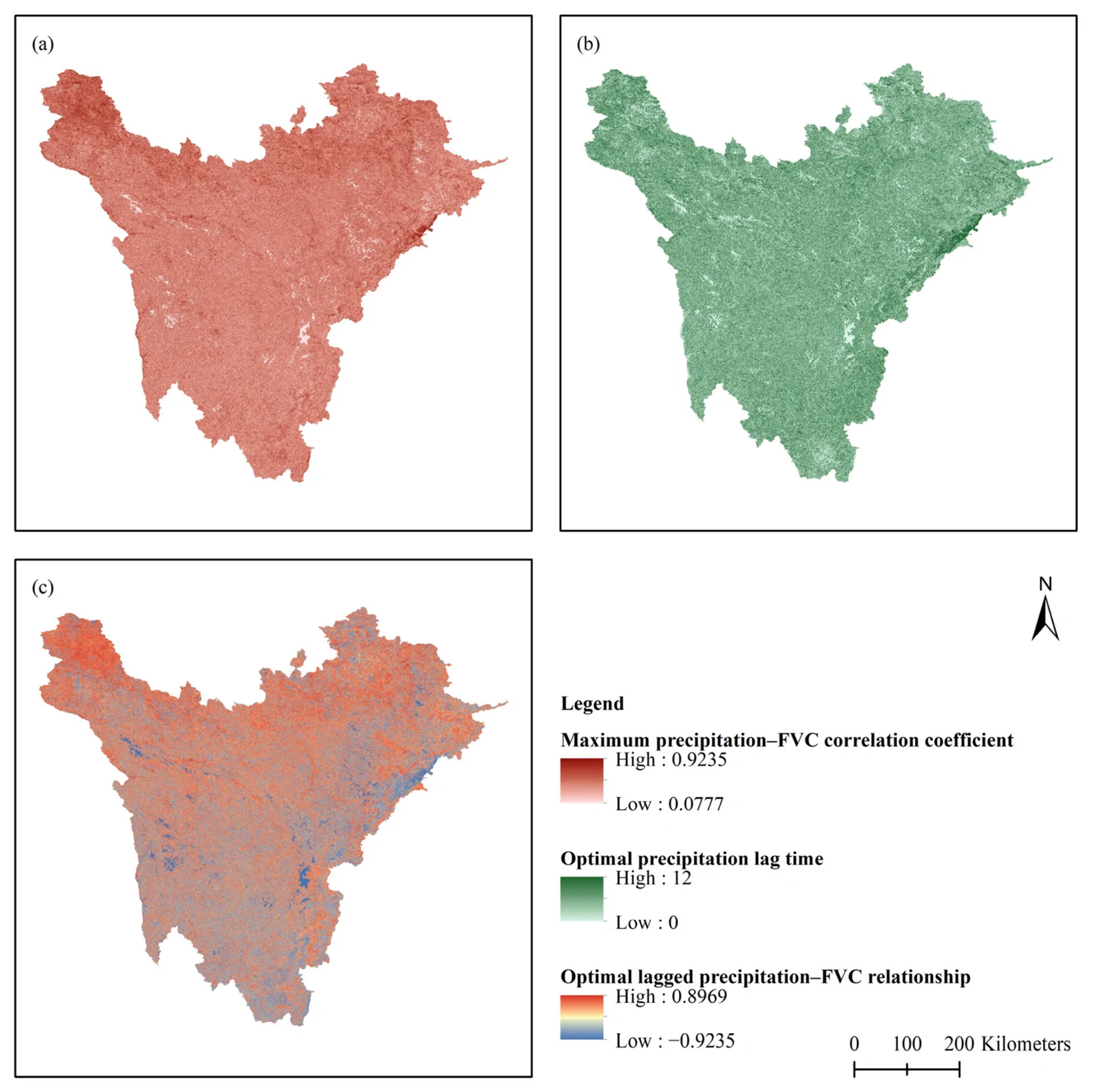
Lagged associations between precipitation variability and vegetation growth across the Western Sichuan Plateau. (**a**) Maximum precipitation–FVC correlation coefficient; (**b**) optimal precipitation lag time; (**c**) optimal lagged precipitation–FVC relationship.

Similar to temperature, precipitation exhibited substantial spatial heterogeneity in its relationship with vegetation dynamics (Figure 5). The maximum absolute correlation coefficient (|r|) between precipitation and FVC ranged from 0.0777 to 0.9235 (Figure 5a), indicating that vegetation responses to water availability varied considerably in different ecological environments. Compared with temperature, precipitation showed slightly stronger overall correlations with FVC, suggesting that water availability represents an important regulating factor for vegetation growth in some regions of the Western Sichuan Plateau. This pattern is particularly relevant for alpine ecosystems where vegetation productivity is strongly constrained by limited soil moisture availability and short growing seasons.

The spatial distribution of optimal precipitation lag time showed that vegetation responses occurred across a broad range of temporal scales, from immediate responses to long-term delayed associations (Figure 5b). The optimal lag period ranged from 0 to 12 months, with long-term lag (7–12 months) representing the dominant category. This indicates that FVC was frequently more strongly associated with antecedent precipitation than with precipitation at the same temporal scale. Under the optimal lag conditions, precipitation–FVC correlation coefficients ranged from −0.9235 to 0.8969 (Figure 5c). Positive correlations were observed in most areas, indicating an association between greater precipitation and higher FVC in these regions. However, negative precipitation–FVC relationships occurred locally, which may be associated with excessive moisture conditions, reduced solar radiation caused by increased cloud cover, or ecosystem-specific responses to precipitation anomalies. Delayed associations between precipitation and FVC may be consistent with processes such as soil-moisture persistence, water redistribution, root water uptake, or physiological adjustment, but these processes are proposed explanations rather than mechanisms demonstrated by the present correlation analysis.

#### 2.2.3. Comparison of Temperature and Precipitation Lagged Associations

To compare the strength of lagged climate–vegetation associations, the maximum absolute Pearson correlation coefficient (|r|) and optimal lag time were jointly analysed for temperature and precipitation. For descriptive purposes, the absolute correlation coefficient (|r|) was classified into five categories of correlation strength: very weak (0–0.2), weak (0.2–0.4), moderate (0.4–0.6), strong (0.6–0.8), and very strong (0.8–1.0), following commonly used interpretive guidelines [33,34]. These categories are used only as descriptive thresholds and do not represent standardised ecological response levels or causal effects. The correlation strength statistics showed that vegetation responses to both temperature and precipitation were dominated by weak and moderate levels (Table 3).

For temperature, weak and moderate strength areas accounted for 44.3956% and 49.8990% of the study area, respectively, together representing 94.2946% of the total area. This indicates that although temperature variability influences vegetation dynamics, most ecosystems exhibited moderate thermal sensitivity rather than strong dependence. For precipitation, weak and moderate strength areas accounted for 44.0868% and 50.4102%, respectively, together accounting for 94.4970% of the study area. Compared with temperature, precipitation showed a slightly higher proportion of moderate-response areas, suggesting that water availability may exert a relatively stronger regulatory effect on vegetation growth in some regions. The proportions of strong and very strong strength were limited for both climatic factors (<3.7%), indicating that vegetation dynamics across most of the Western Sichuan Plateau were not associated with a single climatic variable. Instead, vegetation responses likely resulted from the combined effects of thermal conditions, water availability, terrain constraints, and ecosystem properties.

Analysis of the optimal lag time indicates that both temperature and precipitation showed lagged associations with FVC (Table 4). Long-term lags (7–12 months) were the primary pattern for these two climate variables, accounting for 45.6600% of the area for temperature and 45.0768% for precipitation. Short-term lags accounted for 24.7036% and 21.7752% of the area for temperature and precipitation, respectively, while medium-term lags represented 19.3103% and 23.4553%. Immediate associations were the least common category, accounting for 10.3260% for temperature and 9.6927% for precipitation. These results indicate that FVC was more frequently associated with antecedent climatic conditions than with instantaneous climate variability. The predominance of long-term lags highlights the prevalence of lagged climate–vegetation associations in the study area. The observed lag patterns may be consistent with cumulative thermal conditions and persistent water availability, but the present correlation analysis does not directly demonstrate these underlying mechanisms.

Although both climatic factors exhibited similar lag patterns, the underlying mechanisms cannot be distinguished directly from the correlation analysis. Temperature showed a slightly higher proportion of long-term lag responses (45.6600%) than precipitation (45.0768%), which may be consistent with the influence of cumulative heat availability in high-elevation ecosystems. Precipitation-related lags were more strongly expressed at the medium-term scale (23.4553%) than temperature (19.3103%), which may reflect delayed changes in water availability. Processes such as thermal accumulation, soil moisture storage, water redistribution, root uptake, and plant physiological regulation are possible explanations for these lagged associations, rather than mechanisms demonstrated by the present analysis. Further process-based observations would be required to test these interpretations.

### 2.3. Nonlinear Environmental Associations with FVC Spatial Variation Based on XGBoost–SHAP

#### 2.3.1. Performance Comparison of XGBoost Models with Different Environmental Variables

To assess the contributions of different environmental variables to the spatial variability of FVC, three XGBoost models with gradually increasing environmental complexity were constructed: (1) the climate model, which included only climate variables; (2) the climate–terrain model, which integrated climate and topography variables; and (3) the full ecological model, which included climate, topography, and land-cover information. The performance of the three XGBoost models, evaluated using spatially separated testing samples, is presented in Table 5. Because several predictors are static or relatively stable over the study period, these models primarily characterise spatial environmental heterogeneity rather than directly explaining interannual FVC trends.

The climate-only model achieved an R^2^ value of 0.2181, with RMSE and MAE values of 0.2650 and 0.2060, respectively. Although climate variables captured part of the vegetation variability, their explanatory power remained relatively limited, suggesting that climatic conditions alone cannot fully explain the significant spatial heterogeneity of vegetation coverage in this alpine region. After incorporating topographic variables (DEM, slope, and aspect), the climate–terrain model showed a substantial improvement in predictive performance, with R^2^ increasing to 0.5716, while RMSE and MAE decreased to 0.1961 and 0.1451, respectively. This considerable improvement indicates that topography-related environmental variables provide important information for representing vegetation distribution in alpine landscapes. Further integration of land-cover information resulted in the highest predictive performance. The full ecological model achieved an R^2^ of 0.6393, with RMSE and MAE values reduced to 0.1800 and 0.1263, respectively. The progressive improvement in model performance indicates that terrain and land-cover information provided additional explanatory information beyond climate variables for representing the spatial variability of FVC. Overall, the model comparison supports a hierarchical environmental association structure in the spatial variation in FVC, in which climate conditions provide background information while topographic gradients and ecosystem composition further characterise regional vegetation heterogeneity.

#### 2.3.2. Relative Importance of Environmental Variables Based on SHAP Analysis

**Figure 6:**
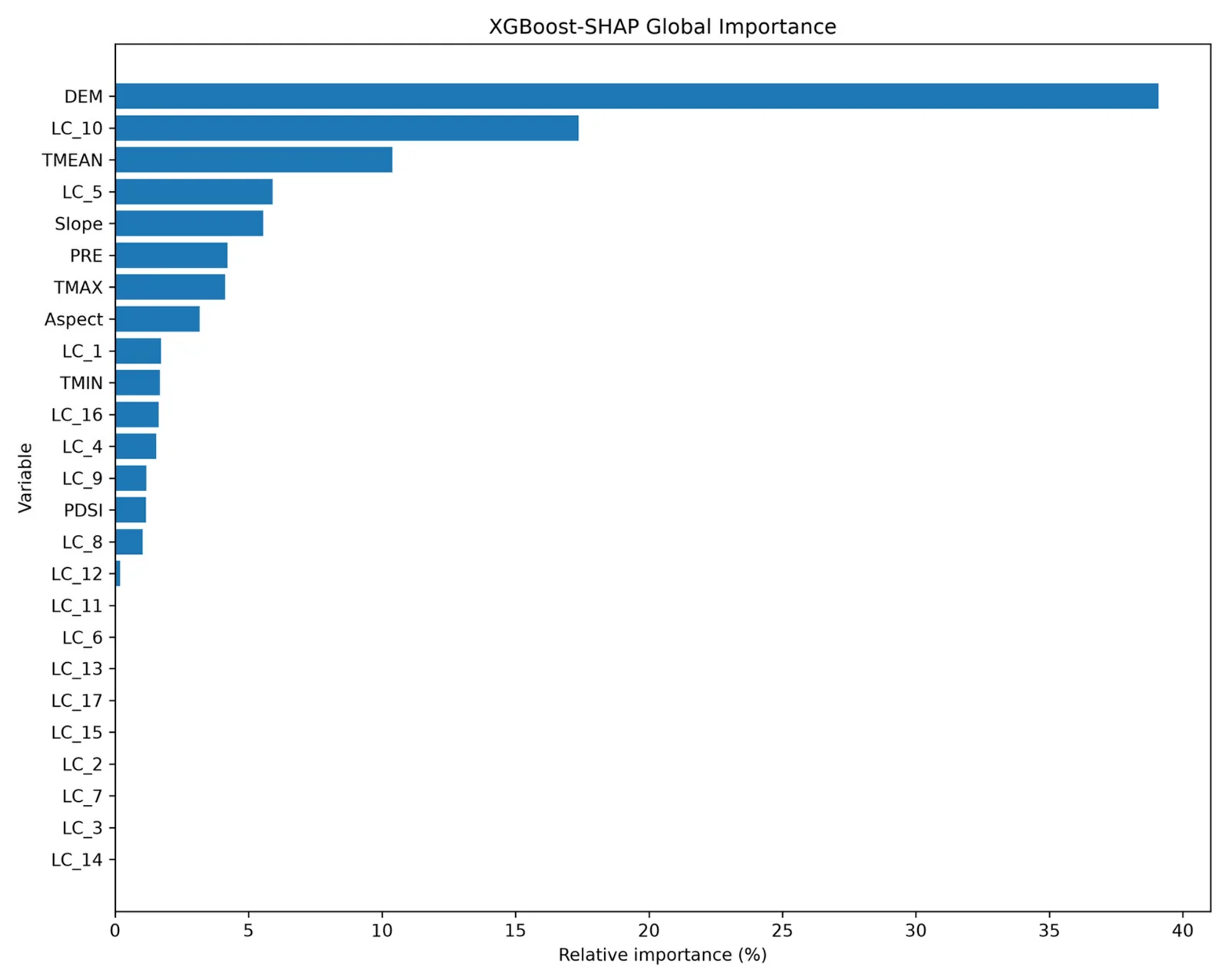
Relative contributions of environmental predictors based on mean absolute SHAP values.

The SHAP analysis revealed substantial differences in the relative contributions of individual predictors to the XGBoost predictions (Figure 6). DEM showed the highest mean absolute SHAP contribution, while several land-cover categories and climatic and topographic variables also contributed to the model predictions. Overall, topographic, land-cover, and climatic variables provided different amounts of explanatory information for representing the spatial heterogeneity of FVC.

Among all environmental variables, altitude (DEM) exhibited the highest mean absolute SHAP contribution (39.0853%). This result indicates that elevation-related spatial information contributed substantially to the model representation of FVC variability. However, because the predictors originated from datasets with different native spatial resolutions, the SHAP ranking should be interpreted as the relative contribution of the available environmental information rather than as a resolution-independent measure of ecological importance. The relatively high contribution of DEM may partly reflect its ability to represent fine-scale topographic heterogeneity across the mountainous landscape. Elevation is also spatially associated with several environmental gradients, including temperature, moisture availability, and vegetation distribution. Therefore, the high SHAP contribution of DEM should not be interpreted as evidence that elevation alone exerts a stronger ecological effect than climate variables.

Several land-cover categories exhibited relatively high SHAP contributions, indicating that ecosystem-type information provided substantial explanatory information for the spatial representation of FVC. Among them, LC_10 (grasslands) showed the highest relative importance among land-cover classes (17.3644%), indicating that grassland-related spatial information contributed substantially to the representation of regional FVC patterns. LC_5 (mixed forests) contributed 5.9048%, also showing considerable explanatory power. However, because some land-cover classes are intrinsically associated with vegetation status, their high SHAP contributions should not be interpreted as evidence of independent causal effects on FVC. The contribution of land-cover variables may partly reflect their ability to represent spatially explicit ecosystem structure at a finer scale than the climate datasets. Consequently, relatively high SHAP contributions of land-cover categories should be interpreted as evidence that ecosystem-type information improves the representation of FVC spatial heterogeneity, rather than as evidence of stronger intrinsic ecological effects than climatic variables.

Among climatic variables, TMEAN had the highest contribution, with a relative importance of 10.3859%, indicating that thermal conditions remain an important climatic limiter for alpine vegetation growth. TMAX and PRE contributed 4.1220% and 4.2159%, respectively, while TMIN and PDSI showed relatively lower contributions (1.6794% and 1.1600%, respectively). Overall, the sum of topographic variables (DEM, slope, and aspect: 47.7919%) was higher than that of land-cover variables (approximately 30.6450%) and climatic variables (approximately 21.5632%), confirming that topographic heterogeneity, together with ecosystem type, plays a dominant role in regulating the spatial variability of FVC on the Western Sichuan Plateau.

#### 2.3.3. Nonlinear Response Characteristics of Dominant Environmental Factors

**Figure 7:**
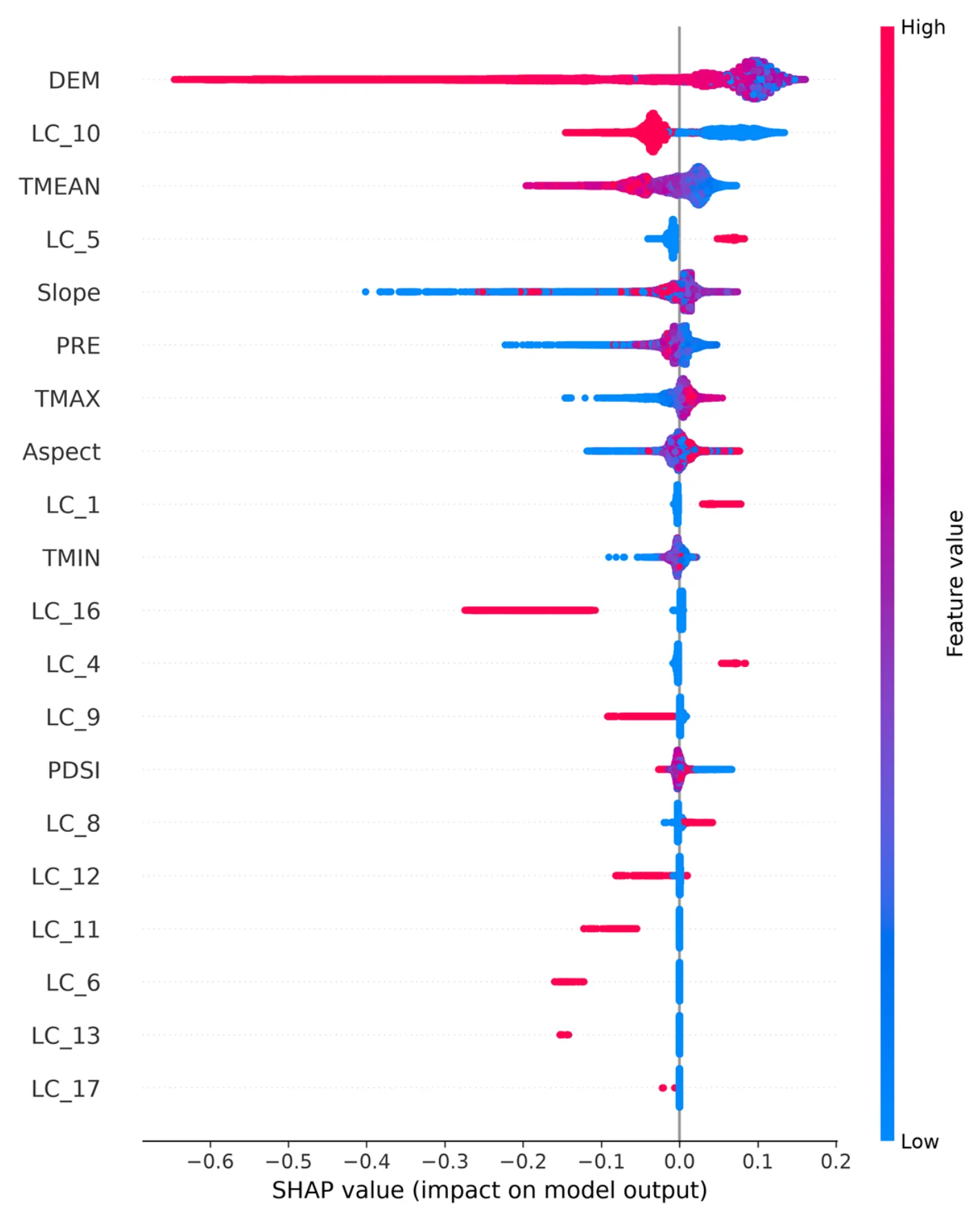
SHAP dependence patterns between dominant environmental variables and FVC variability.

The SHAP dependence patterns further revealed nonlinear relationships between dominant environmental variables and FVC variability (Figure 7). Unlike traditional linear models, the XGBoost-SHAP framework indicates that vegetation responses vary significantly along environmental gradients.

Among all predictor variables, DEM exhibited the widest range in SHAP values and made the largest contribution to the modelled spatial variation in FVC. Higher elevation values were generally associated with negative SHAP contributions, while lower elevation conditions were more frequently associated with positive contributions. This pattern indicates that elevation-related spatial gradients were strongly represented in the model predictions. LC_10 (grasslands) was mainly associated with negative SHAP contributions, indicating that grassland-related spatial information generally corresponded to relatively lower FVC levels compared with other land-cover categories. The SHAP response of TMEAN demonstrated a nonlinear association with FVC. Although moderate temperature conditions were generally associated with positive contributions, higher temperature conditions showed partially negative SHAP effects. This pattern suggests that the model captured nonlinear spatial associations between thermal conditions and FVC. Precipitation exhibited an overall positive SHAP response pattern, with higher precipitation values generally associated with positive contributions to FVC prediction.

#### 2.3.4. Period-Specific Consistency of Environmental Associations with FVC

**Figure 8:**
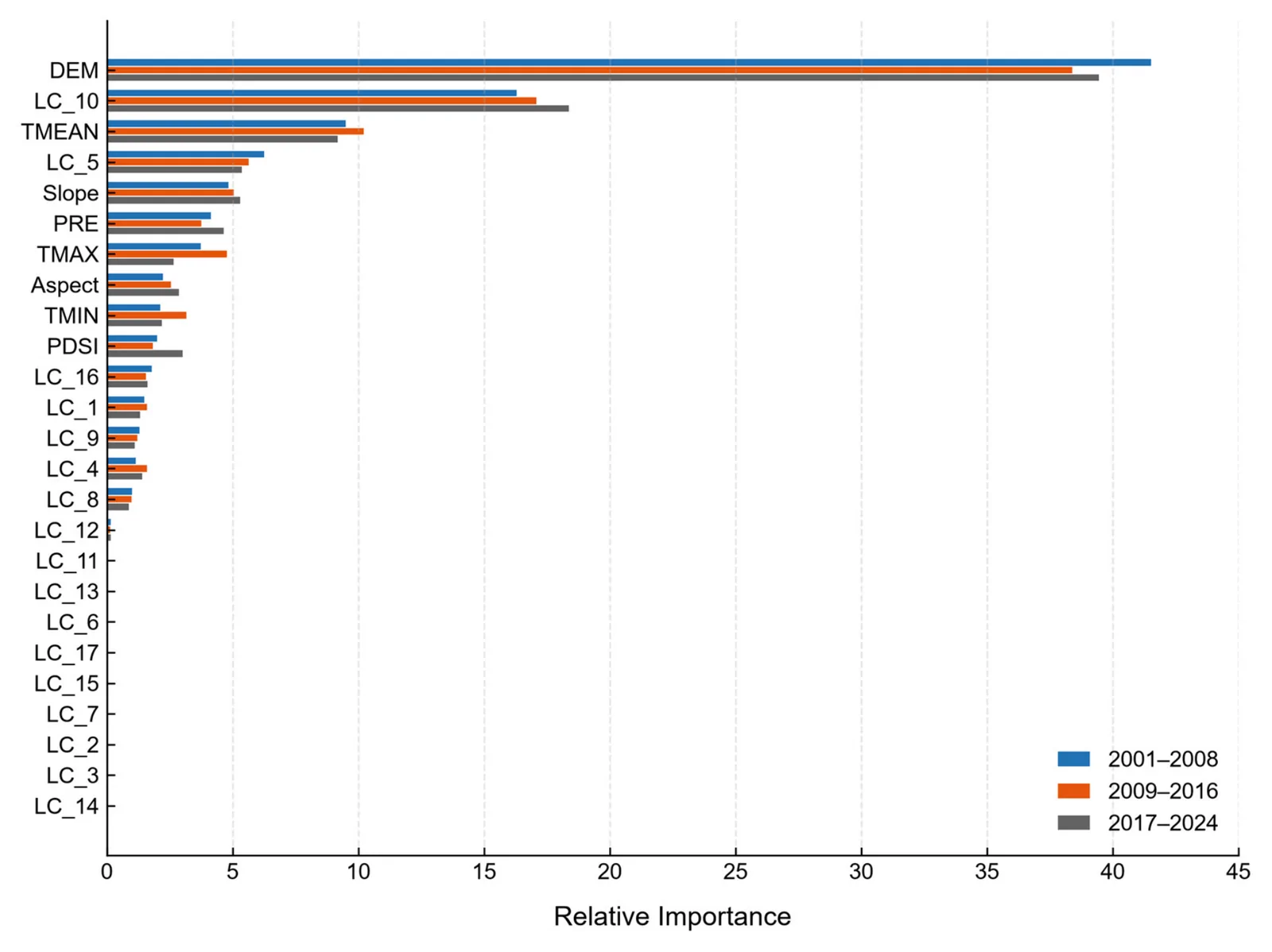
Relative importance of environmental variables across different periods.

To examine whether the environmental associations represented by the XGBoost models varied across the study period, the study period was divided into three stages: 2001–2008, 2009–2016, and 2017–2024. Separate XGBoost models were constructed for each period, and SHAP-based variable contributions were compared to characterise period-specific differences in model attribution (Table 6; Figure 8). The R^2^ values for 2001–2008, 2009–2016, and 2017–2024 were 0.6515, 0.6424, and 0.6490, respectively. The RMSE values were 0.1783, 0.1789, and 0.1764, while MAE values were 0.1268, 0.1258, and 0.1225, respectively. Model performance was broadly comparable among the three periods, indicating that the selected environmental variables provided similar levels of predictive information for the spatial variability of FVC across the three time windows.

DEM consistently exhibited the highest mean absolute SHAP contribution among the predictors, with relative importance values of 41.5501%, 38.4032%, and 39.4698% during 2001–2008, 2009–2016, and 2017–2024, respectively. Although its contribution fluctuated slightly among periods, DEM remained the dominant predictor of spatial FVC variation in the three period-specific models, indicating the persistent importance of elevation-related spatial gradients in representing FVC heterogeneity. LC_10 (grasslands) consistently ranked among the most important land-cover variables, with relative importance increasing from 16.3231% during 2001–2008 to 17.1054% during 2009–2016 and 18.3932% during 2017–2024. This variation indicates differences in the contribution of grassland-related spatial information to model predictions among periods. TMEAN maintained a relatively stable contribution across periods, with relative importance values of 9.5254%, 10.2364%, and 9.2084%, respectively. PRE importance values changed from 4.1531% to 3.7822% and then increased to 4.6619%, while PDSI contributions changed from 2.0201% to 1.8437% and then rose to 3.0459% across the three periods. These variations indicate that the contribution of precipitation- and drought-related information to model predictions differed among periods.

The XGBoost–SHAP analysis provides complementary information on the environmental factors associated with the spatial variability of FVC across the Western Sichuan Plateau. Unlike the Sen–MK trend analysis, which directly quantifies temporal changes in FVC, the XGBoost framework primarily characterises spatial differences among locations based on climatic, topographic, and land-cover predictors.

## 3. Discussion

### 3.1. Spatiotemporal Characteristics and Ecological Implications of Vegetation Dynamics

Vegetation dynamics in alpine ecosystems are determined by the interaction among climatic factors, topographic gradients, and ecosystem characteristics [2,14]. In this study, temporal trend analysis showed a significant increase in FVC during 2001–2024, while the spatial distribution of FVC exhibited substantial heterogeneity across the Western Sichuan Plateau. These temporal and spatial patterns provide complementary information on vegetation change at the regional scale. The increasing trend of FVC may be related to the combined effects of climate warming and ecological conservation measures [11,35]. As a key ecological barrier in the upper reaches of the Yangtze River, the Western Sichuan Plateau has undergone large-scale ecological restoration and conservation efforts in recent decades [12,36].

The spatial distribution of vegetation changes indicates that both improvement and degradation of vegetation coexist on the Western Sichuan Plateau, which is consistent with the findings of previous studies [4]. Vegetation improvement is primarily observed in the eastern and southeastern regions, where significant and non-significant improvement together accounted for 63.6220% of the study area, while degradation is more common in the western and high-altitude areas, where significant and non-significant degradation together accounted for 23.4809%. This is due to the fact that vegetation changes depend primarily on local environmental conditions rather than following a uniform regional trend [14,24]. In the context of global climate change, alpine ecosystems are showing spatially differentiated responses [2,7]. Regions with relatively favourable environmental conditions may benefit from rising temperatures and ecological restoration, while high-altitude and environmentally fragile areas remain vulnerable to climate-related pressures. The coexistence of widespread greening and localised degradation suggests that the vegetation response of the Western Sichuan Plateau is not spatially uniform but is regulated by the combined effects of topography, ecosystem type, and hydrothermal conditions.

### 3.2. Possible Explanations for Lagged Climate–FVC Associations

A key finding of this study is that vegetation responses to both temperature and precipitation were characterised by lagged climate–vegetation associations, with long-term lag periods (7–12 months) representing the largest proportion for both climatic variables. Specifically, long-term lag responses accounted for 45.6600% of the study area for temperature and 45.0768% for precipitation (Table 4). Because the analysis was conducted using seasonally adjusted climate anomalies, these results indicate delayed statistical associations between antecedent climate conditions and subsequent FVC rather than direct evidence of physiological lagged climate–vegetation associations.

In high-altitude environments, vegetation growth is strongly limited by thermal conditions [7]. Temperature not only influences current photosynthetic activity but can also be related to cumulative processes, including snowmelt timing, soil temperature recovery, plant dormancy release, and growing-season initiation [2,14]. Therefore, the dominance of long-term temperature lags (45.6600%) may be consistent with the role of accumulated thermal conditions in alpine ecosystems. Previous-year thermal conditions could potentially influence subsequent vegetation growth through overwinter survival and early-season growth potential [4,25], but this interpretation cannot be demonstrated directly from the correlation analysis. The remaining distribution of short-term (24.7036%) and medium-term (19.3103%) lags, together with immediate associations (10.3260%), further indicates substantial spatial variation in the temporal association between temperature and FVC.

The long-term precipitation lag occupied 45.0768% of the study area, suggesting that antecedent precipitation conditions were frequently associated with subsequent FVC. Medium-term precipitation lags accounted for 23.4553%, which was higher than the corresponding temperature medium-term lag (19.3103%), while short-term precipitation lags accounted for 21.7752%, lower than the temperature short-term lag (24.7036%). The observed pattern may be consistent with delayed changes in water availability. Processes such as infiltration, storage, redistribution, and root uptake provide possible explanations for delayed precipitation–FVC associations [14], but they were not directly measured or tested in this study. Compared with temperature, precipitation showed a higher proportion of moderate-correlation areas (50.4102% vs. 49.8990%) and a slightly lower proportion of weak-correlation areas (44.0868% vs. 44.3956%), indicating that water availability was statistically associated with FVC at somewhat greater correlation strength in some regions [22]. Under climate warming scenarios, enhanced evapotranspiration demand may increase ecosystem water stress and potentially strengthen dependence on precipitation supply [3,6,10].

Although temperature and precipitation showed similar temporal association patterns, the present analysis does not allow their underlying physiological mechanisms to be distinguished directly. Temperature showed a slightly higher proportion of long-term lag responses (45.6600%) than precipitation (45.0768%), which may be consistent with cumulative heat availability. In contrast, precipitation-related lag effects were more strongly expressed at the medium-term scale (23.4553%) than temperature (19.3103%), which may be consistent with delayed water supply conditions and soil moisture persistence [4]. Therefore, the observed lagged associations are compatible with combined thermal and water-availability processes, but they should not be interpreted as direct evidence of specific causal mechanisms.

### 3.3. Spatial Environmental Associations with FVC Revealed by XGBoost–SHAP

Traditional correlation-based methods often simplify the relationship between vegetation and the environment to a linear association, which limits their ability to capture nonlinear ecological dynamics [31]. The XGBoost-SHAP framework applied in this study revealed a hierarchical environmental control structure underlying the spatial variability of FVC on the Western Sichuan Plateau. The model comparison indicates that climate variables can only explain a portion of the spatial variability in FVC (R^2^ = 0.2181). Incorporating topographic information substantially improved model performance (R^2^ = 0.5716), while further inclusion of land-cover information increased explanatory power to R^2^ = 0.6393. This result indicates that vegetation distribution is not simply determined by climatic variability but is strongly regulated by topographic and ecosystem characteristics [20].

Elevation (DEM) has always been recognised as the important environmental factor, accounting for 39.0853% of relative importance in the full ecological model. The dominant role of elevation reflects its integrated representation of multiple ecological gradients, including temperature decrease, radiation variation, soil development, and growing-season limitation [37,38]. As a fundamental environmental factor, elevation controls vegetation establishment and productivity in alpine regions [11,29]. The high contribution of DEM should therefore be interpreted in the context of its strong spatial association with multiple environmental gradients, including temperature, precipitation, vegetation types, and growing-season constraints. In a mountainous ecosystem, elevation can serve as an integrated spatial indicator of several ecological gradients rather than representing a single isolated causal factor.

Land-cover characteristics represented another major source of spatial information in the model. LC_10 (grasslands) showed the second-largest contribution (17.3644%) and remained important across all periods. Its relative SHAP contribution varied from 16.3231% (2001–2008) to 18.3932% (2017–2024), but this pattern should not be interpreted as evidence of increasing ecological importance. Land-cover variables were included primarily as indicators of ecosystem structure and surface characteristics rather than as independent causal drivers of FVC. This distinction is especially important because land-cover classification is conceptually related to vegetation status and may contain information overlapping with the FVC response variable. However, land-cover information remains useful for representing broad ecosystem differences and spatial heterogeneity across the Western Sichuan Plateau. Therefore, the SHAP contribution of land-cover variables should be interpreted as information describing spatial ecosystem structure rather than as evidence of temporal changes in vegetation or independent causal effects.

Although climatic variables showed lower relative importance compared with topography and land-cover type, thermal conditions remained an important component of the spatial environmental information represented by the model. During all periods, the contribution of TMEAN persisted at approximately 10%, indicating that temperature-related information contributed consistently to the representation of FVC spatial heterogeneity. The increasing contribution of precipitation and drought-related variables in recent periods suggests differences in the contribution of hydroclimatic information among spatially separated samples [3,6,35]. Enhanced atmospheric evaporative demand may increase ecosystem dependence on available moisture, especially in high-elevation grassland systems [7,22]. In addition, TMAX showed a notable decline in relative importance from 4.7886% during 2009–2016 to 2.6704% during 2017–2024, whereas TMIN remained relatively low across all periods (2.1506%, 3.1827%, and 2.2126%). In summary, the XGBoost–SHAP results indicate that spatial FVC variation is associated with topographic constraints, ecosystem structure, and hydrothermal gradients. The sum of topographic variables (47.7919%) was higher than that of land-cover variables (30.6450%) and climatic variables (21.5632%), supporting the importance of these environmental gradients in representing spatial FVC heterogeneity. However, these results should not be interpreted as evidence that the identified variables directly control interannual FVC trends.

### 3.4. Implications for Alpine Ecosystem Management Under Climate Change

The Western Sichuan Plateau represents a climate-sensitive alpine ecosystem where vegetation stability is essential for maintaining regional ecological security and water conservation functions [35]. Although vegetation coverage exhibited a significant increasing trend during 2001–2024 (0.0018 yr^−1^, R^2^ = 0.5040, *p* < 0.001), both vegetation improvement and degradation occurred simultaneously in different areas, indicating that the ecosystem has responded differently to changing environmental conditions. The XGBoost-SHAP analysis revealed a strong controlling effect of topography, indicating that high-altitude ecosystems are in a vulnerable state due to limited thermal resources and a short growing season [11]. Therefore, conservation strategies should prioritise high-altitude regions that remain environmentally fragile and are at high risk of vegetation degradation. In the eastern regions, where productivity is relatively high, maintaining ecosystem integrity and preventing disturbances may be important. In contrast, in high-altitude western regions, conservation measures focused on soil moisture conservation, grazing management, and ecosystem restoration may be considered [6].

### 3.5. Limitations of the Study

A limitation of the regional percentile approach is that the P5 and P95 NDVI thresholds were estimated across the entire study area rather than separately within elevation zones or land-cover classes. Because the Western Sichuan Plateau encompasses substantial gradients in vegetation structure, elevation, and surface conditions, ecosystem-specific thresholds could potentially better represent local vegetation and background conditions. Future studies could further evaluate the robustness of FVC estimates using stratified NDVI reference thresholds based on elevation or ecosystem type.

The interpretation of variable importance should also account for differences in the native spatial resolutions of the environmental datasets. Although all predictors were harmonised to a common 250 m analysis grid, their original spatial supports remained different. Climate variables at approximately 11 km resolution represent broad-scale hydrothermal conditions, whereas DEM and land-cover products contain relatively finer-scale spatial information. Consequently, the relatively high SHAP contributions of DEM and land-cover variables may partly reflect their capacity to capture fine-scale spatial heterogeneity that is not represented by the coarser climate datasets. The SHAP ranking should therefore be interpreted as the relative contribution of available environmental information within the specified modelling framework, rather than as a direct comparison of the intrinsic ecological strength of different environmental factors. Future analyses using predictors harmonised to comparable effective spatial scales could provide a more rigorous assessment of scale-dependent environmental contributions.

## 4. Materials and Methods

### 4.1. Study Area

**Figure 9:**
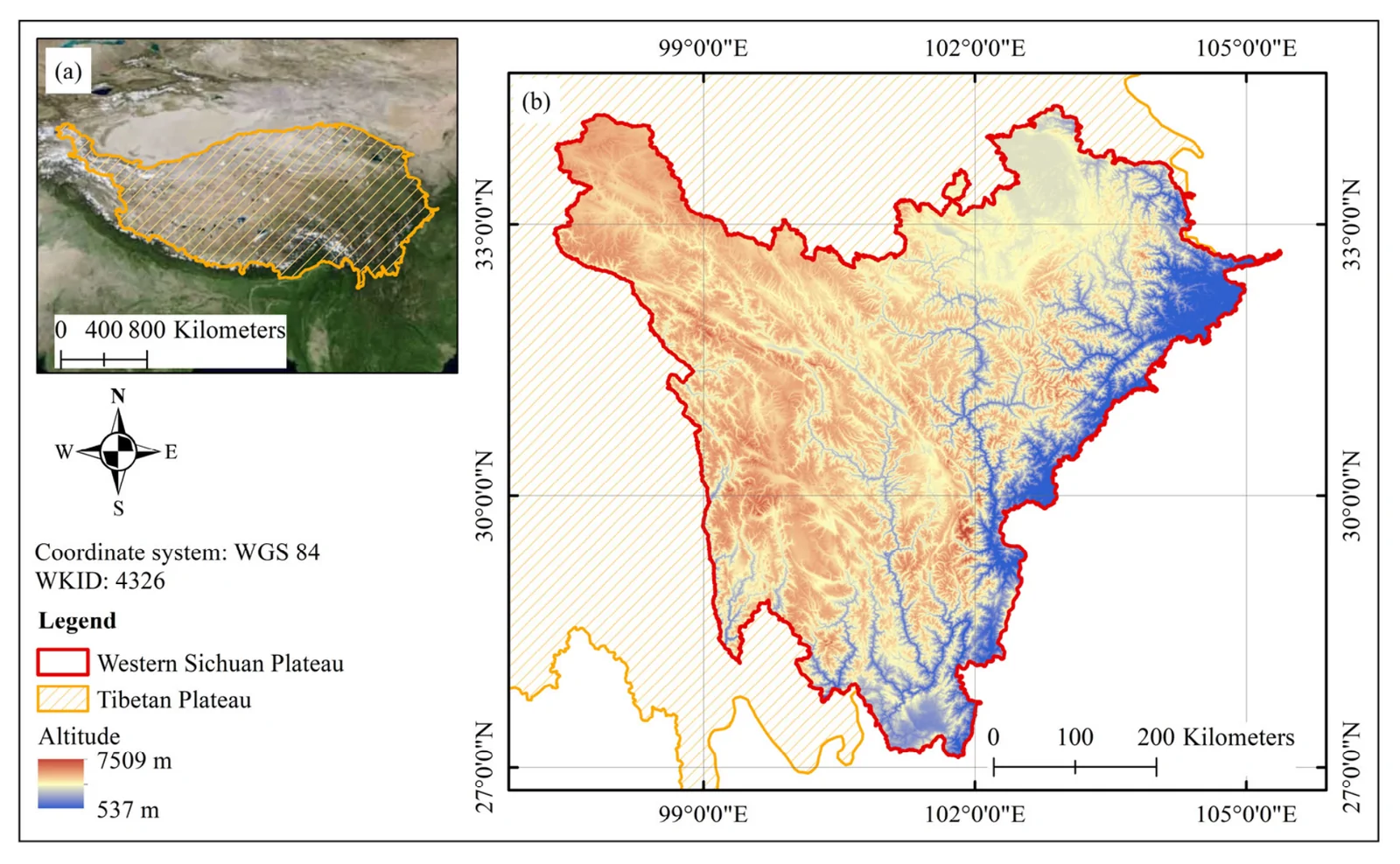
Location of the study area. (**a**) Location of the Western Sichuan Plateau on the eastern Tibetan Plateau; (**b**) spatial distribution of elevation across the Western Sichuan Plateau.

The Western Sichuan Plateau, located in the Eastern Tibetan Plateau (Figure 9a), is one of the world’s most important alpine ecosystems. The region is characterised by high elevation, complex and varied topography, and significant hydrothermal gradients, which have resulted in a diverse ecological environment and vegetation pattern (Figure 9b). As an important ecological barrier and water conservation region in the upper reaches of the Yangtze River, the Western Sichuan Plateau plays a critical role in regional water regulation, biodiversity conservation, and ecological security maintenance. The study area contains a variety of alpine ecosystems, including alpine meadows, alpine grasslands, shrublands, and forest ecosystems [39]. Due to the combined constraints of low temperature, short growing seasons, and fragile environmental conditions, vegetation conditions in this region are strongly shaped by climate variability, topographic gradients, and ecosystem characteristics [40,41]. The topography exhibits strong spatial heterogeneity, with elevation gradually increasing from the eastern mountains to the western plateau, forming distinct vertical vegetation zones and environmental gradients. In this study, the spatial boundary of the Western Sichuan Plateau was used to define the study area. All remote sensing preprocessing, spatial analysis, and ecological modelling were conducted on the Google Earth Engine (GEE) cloud computing platform (https://developers.google.com/earth-engine, accessed on 29 July 2026). The total area of the study region is approximately 383,006.80 km^2^.

### 4.2. Data Sources

This study integrated remote sensing, climate, topography, and land-cover datasets to characterise FVC dynamics and examine spatial environmental associations. The datasets used in this study are summarised in Appendix A Table A1.

#### 4.2.1. MODIS NDVI Data

Vegetation dynamics were characterised using the MODIS Terra Vegetation Index product (MOD13Q1, V 6.1). The MOD13Q1 product provides normalised difference vegetation index (NDVI) observations with a spatial resolution of 250 m and a temporal interval of 16 days, and has been widely applied in regional-scale vegetation monitoring studies [29]. The MODIS NDVI dataset was obtained from the GEE (https://developers.google.com/earth-engine/datasets/catalog/MODIS_061_MOD13Q1, accessed on 29 July 2026). The original NDVI values (${\mathrm{NDVI}}_{\mathrm{original}}$) were converted using the official scale factor provided by the dataset documentation:

(1) $$\mathrm{NDVI}\;=\;{\mathrm{NDVI}}_{\mathrm{original}}\;\times \;0.0001$$

The study period covered 2001–2024. All NDVI images were first clipped using the study boundary and subsequently used for FVC estimation and long-term vegetation change analysis.

#### 4.2.2. Climate Data

Climate variables were obtained from the ERA5-Land Monthly Aggregated—ECMWF Climate Reanalysis dataset (https://developers.google.com/earth-engine/datasets/catalog/ECMWF_ERA5_LAND_MONTHLY_AGGR, accessed on 29 July 2026). ERA5-Land provides high-resolution land surface climate information, which has been widely used to study ecosystem responses to climate variability [3,42]. Four climate variables were extracted in this study, including: 2 m air temperature (temperature_2m, TMEAN), minimum 2 m air temperature (temperature_2m_min, TMIN), maximum 2 m air temperature (temperature_2m_max, TMAX), and total precipitation (total_precipitation_sum, PRE). The spatial resolution of ERA5-Land data is approximately 11,132 m.

For ecological analysis, monthly climate variables were aggregated into annual-scale indicators corresponding to the vegetation growing season. Since the ERA5-Land temperature variables are expressed in kelvin (K), the temperature values were converted to degrees Celsius (°C):

(2) T(℃) = T(K) − 273.15 

Precipitation values were converted from metres (m) to millimetres (mm):

(3) PRE(mm) = PRE(m) × 1000

In addition, the Palmer Drought Severity Index (PDSI) derived from the TerraClimate dataset (https://developers.google.com/earth-engine/datasets/catalog/IDAHO_EPSCOR_TERRACLIMATE, accessed on 29 July 2026) was used to characterise regional moisture conditions. The TerraClimate dataset has a spatial resolution of approximately 4638.3 m [43]. Monthly PDSI values were averaged annually and incorporated into the environmental-association analysis to represent drought-related environmental stress.

#### 4.2.3. Topographic and Land-Cover Datasets

Topographic information was obtained from the SRTM digital elevation model (https://developers.google.com/earth-engine/datasets/catalog/USGS_SRTMGL1_003, accessed on 29 July 2026). The SRTM DEM dataset has a spatial resolution of approximately 30 m. Based on the DEM data, three topographic variables were derived, including elevation (DEM), slope, and aspect, which were used to characterise terrain-related environmental associations.

Land-cover variables were derived from the MODIS land-cover product (MCD12Q1, V 6.1, https://developers.google.com/earth-engine/datasets/catalog/MODIS_061_MCD12Q1, accessed on 29 July 2026) and included as categorical descriptors of ecosystem type. These variables were used to represent broad differences in ecosystem structure and surface characteristics across the study region. Given the conceptual relationship between land-cover classification and vegetation cover, land-cover contributions were not interpreted as direct causal effects on FVC. The MCD12Q1 has a spatial resolution of 500 m and provides annual land-cover classification based on the International Geosphere-Biosphere Programme (IGBP) classification system (Table A2). Annual land-cover maps from 2001 to 2024 were extracted and incorporated as categorical predictors in the environmental association analysis. Based on the original land-cover values (1–17), the land-cover categories were re-coded as LC_1–LC_17. This allowed land-cover types to be regarded as categorical variables rather than continuous numerical predictor variables in later machine-learning analyses.

### 4.3. Estimation of FVC

The FVC is an important quantitative indicator for characterising vegetation status and ecosystem structure, and has been widely applied in regional-scale vegetation monitoring studies. In this study, FVC was estimated using the pixel dichotomy model based on NDVI. This model assumes that a remote sensing pixel is composed of vegetation and non-vegetation components, and the proportion of vegetation within each pixel can be derived from the spectral difference between vegetation and background surfaces. The pixel dichotomy model was expressed as follows [35,44]:

(4) $$\mathrm{FVC}\;=\;\frac{\mathrm{NDVI}-{\mathrm{NDVI}}_{\mathrm{soil}}}{{\mathrm{NDVI}}_{\mathrm{veg}}-{\mathrm{NDVI}}_{\mathrm{soil}}}$$

where FVC represents fractional vegetation cover, ${\mathrm{NDVI}}_{\mathrm{soil}}$ represents the NDVI value of the bare soil pixels, and ${\mathrm{NDVI}}_{\mathrm{veg}}$ represents the NDVI value of the fully vegetated pixels. The calculated FVC values were constrained within the range of 0–1.

#### 4.3.1. Annual Growing-Season NDVI Composite

Vegetation growth on the Western Sichuan Plateau is strongly constrained by low temperatures and a short growing season. Therefore, the period from May to September was selected as the vegetation growing season based on regional climatic conditions and vegetation phenology. To minimise the influence of cloud cover, atmospheric effects, and short-term anomalies, the maximum value composite (MVC) method was applied to generate annual growing-season NDVI composites [14,45]:

(5) $${\mathrm{NDVI}}_{y}\;=\;\max({\mathrm{NDVI}}_{i})$$

where ${\mathrm{NDVI}}_{y}$ represents the annual growing-season maximum NDVI in year y, and ${\mathrm{NDVI}}_{i}$ represents the NDVI observation during each composite period within the growing season.

Based on MOD13Q1 NDVI data from 2001 to 2024, annual maximum NDVI images for the growing season were generated for each year. These images were subsequently used for annual FVC estimation.

#### 4.3.2. Fixed-Reference NDVI Endmembers

Traditional FVC estimation methods commonly use constant NDVI thresholds for vegetation and soil pixels. However, fixed thresholds may not adequately represent the overall background conditions of a heterogeneous region, while annually recalculated thresholds may introduce a temporally varying reference scale that complicates the interpretation of long-term trends. To address both issues, this study employed a fixed-reference endmember approach based on a common NDVI distribution over the entire study period.

For the entire study period, the long-term mean growing-season NDVI was calculated as:

(6) $${\mathrm{NDVI}}_{Mean}\;=\;\frac{1}{n}\sum_{i=1}^{i=n}{\mathrm{NDVI}}_{i}$$

where ${\mathrm{NDVI}}_{i}$ represents the annual growing-season maximum NDVI in year i, and n = 24. The 5th and 95th percentiles of this long-term mean NDVI distribution were then extracted as the fixed soil and vegetation endmembers:

(7) $${NDVI}_{soil}=P5({NDVI}_{Mean})$$

(8) $${NDVI}_{veg}=P95({NDVI}_{Mean})$$

where P5 represents the 5th percentile and P95 represents the 95th percentile of the common NDVI distribution derived from the entire 2001–2024 study period. These fixed endmembers were applied consistently to all annual FVC estimates, avoiding an annually changing reference scale that could suppress or distort interannual FVC variability. The 5th percentile NDVI value was considered representative of the low-vegetation or bare-soil background, whereas the 95th percentile NDVI value represented dense vegetation conditions.

#### 4.3.3. Multi-Year Mean FVC

To characterise the overall FVC pattern during the study period, the multi-year mean FVC was calculated using annual growing-season FVC values from 2001 to 2024:

(9) $${\mathrm{FVC}}_{\mathrm{mean}}\;=\;\frac{1}{n}\sum_{i\;=\;1}^{n}{\mathrm{FVC}}_{i}$$

where ${\mathrm{FVC}}_{\mathrm{mean}}$ represents the multi-year mean FVC, ${\mathrm{FVC}}_{i}$ represents the annual growing-season FVC value, and n = 24. The resulting multi-year average FVC was used to analyse the spatial distribution characteristics and patterns of FVC in the Western Sichuan Plateau.

### 4.4. Vegetation Trend Analysis

To quantify long-term vegetation change trends during 2001–2024, the Sen’s slope estimator combined with the Mann–Kendall (MK) significance test was applied to the annual growing-season FVC time series. The Sen-MK method is a non-parametric statistical approach that has been widely used in long-term ecological and environmental studies because it is insensitive to extreme values and does not require the time series to follow a normal distribution [21,24,46].

#### 4.4.1. Sen’s Slope Estimator

Sen’s slope estimator was used to quantify the magnitude and direction of annual FVC changes [47]:

(10) $$Sen’s\;slope\;=\;\mathrm{Median}(\frac{{\mathrm{FVC}}_{j}{-\mathrm{FVC}}_{i}}{j-i}),\;j\;>\;i$$

where ${\mathrm{FVC}}_{i}$ and ${\mathrm{FVC}}_{j}$ represent FVC values in years i and j, respectively. A positive Sen’s slope value indicates an increasing vegetation trend, suggesting vegetation improvement, while a negative value indicates vegetation degradation. The Sen’s slope value was calculated independently for each pixel to generate the spatial distribution of vegetation change rates across the study area.

#### 4.4.2. Mann–Kendall Significance Test

The Mann–Kendall test was used to evaluate the statistical significance of vegetation trends. The MK statistic was calculated as [42]:

(11) $$S\;=\;\sum_{i\;=\;1}^{n-1}\sum_{j\;=\;i+1}^{n}\mathrm{sign}(x_{j}\;-\;x_{i})$$

where $x_{i}$ and $x_{j}$ represent FVC values in years i and j, respectively. The standardised statistic Z was subsequently calculated to determine the significance level of the observed trend. A significance threshold of *p* < 0.05 was adopted to identify statistically significant changes.

Based on the direction of Sen’s slope and MK significance level, FVC trends were classified into five categories: significant improvement (Sen’s slope > 0, *p* < 0.05), non-significant improvement (Sen’s slope > 0, *p* > 0.05), stability (Sen’s slope = 0), non-significant degradation (Sen’s slope < 0, *p* > 0.05), and significant degradation (Sen’s slope < 0, *p* < 0.05). This classification system is used to quantify the spatial distribution and proportion of area for different vegetation change trends.

### 4.5. Climate Lag-Response Analysis

In alpine ecosystems, vegetation growth may be associated not only by current climatic conditions but also with antecedent climatic states, which can produce lagged climate–vegetation associations. Processes such as soil moisture persistence, thermal accumulation, and ecological adaptation are possible explanations for time offsets between climate variability and vegetation conditions. Therefore, this study employed monthly scale lagged correlation analysis to quantify statistical associations between antecedent temperature and precipitation conditions and subsequent FVC in the Western Sichuan Plateau.

The annual growing-season maximum FVC was used as the vegetation response variable. Monthly temperature and precipitation data from ERA5-Land were selected as climatic drivers. To remove the influence of the seasonal cycle, monthly climate anomalies were calculated by subtracting the long-term monthly climatology (2000–2024) from the raw monthly temperature and precipitation data. The lag correlation analysis between growing-season maximum FVC and climate variables was therefore performed using seasonally adjusted anomalies rather than raw monthly values. This avoids spurious lag correlations caused by seasonal phase shifts and co-movement between vegetation phenology and the annual climate cycle.

Considering that climatic impacts on alpine vegetation may persist for an extended period, 13 lag periods ranging from 0 to 12 months were considered. For each pixel, Pearson correlation coefficients between FVC and climatic variables under different lag periods were calculated:

(12) $$r\;=\;\frac{\mathrm{Cov}(X,Y)}{\sigma_{X}\sigma_{Y}}$$

where r represents the Pearson correlation coefficient, Cov(X, Y) represents the covariance between climate variables and FVC, and $\sigma_{X}$ and $\sigma_{Y}$ represent the standard deviations of the corresponding variables.

The lag period corresponding to the maximum absolute correlation coefficient was defined as the optimal lag time:

(13) $${\mathrm{lag}}_{\mathrm{opt}}\;=\;\mathrm{argmax}(\left|r\right|)$$

The corresponding maximum correlation coefficient was defined as:

(14) $$r_{\max}\;=\;\max(\left|r\right|)$$

Finally, three spatial products were generated: (1) spatial distribution of maximum climate–vegetation correlation intensity; (2) spatial distribution of optimal lag time; (3) spatial distribution of correlation direction.

To compare the correlation strength between vegetation and temperature or precipitation, the maximum absolute correlation coefficient ($\left|r\right|$) was classified into five correlation strength categories: very weak (0–0.2), weak (0.2–0.4), moderate (0.4–0.6), strong (0.6–0.8), and very strong (0.8–1.0). In addition, optimal lag times were categorised into four groups: immediate association (0 months), short-term lag (1–3 months), medium-term lag (4–6 months), and long-term lag (7–12 months). These classifications were used as descriptive categories to quantify the spatial distribution of correlation strength and optimal lag time between temperature or precipitation and FVC.

### 4.6. XGBoost–SHAP Framework for Interpreting Environmental Associations with FVC Spatial Variation

Traditional statistical methods usually assume linear relationships and may not fully capture nonlinear associations among environmental variables. Recent advances in machine learning provide opportunities for representing complex relationships among predictors [30,31,32]. In this study, the extreme gradient boosting (XGBoost) algorithm was integrated with the SHapley Additive exPlanations (SHAP) framework to quantify nonlinear associations between climatic, topographic, and land-cover variables and FVC. The XGBoost–SHAP framework was applied as an interpretable machine-learning approach rather than a causal inference model. It was designed to (1) evaluate how different environmental variable combinations explain spatial variation in FVC, and (2) compare the relative contributions of environmental variables to model predictions across the full study period and three sub-periods. Because DEM, slope, aspect, and land-cover characteristics are static or relatively stable over the study period, their SHAP contributions primarily represent spatial environmental heterogeneity rather than direct explanations of interannual FVC trends.

#### 4.6.1. Environmental Variable Selection and Dataset Construction

To characterise the environmental conditions associated with spatial FVC variability, environmental variables were selected from three major categories: climate, topography, and land-cover characteristics. The response variable was annual growing-season maximum FVC. The explanatory variables included climate variables (TMEAN, TMIN, TMAX, PRE, PDSI), topographic variables (DEM, slope, aspect), and land-cover variables (LC). These variables represent climatic conditions, terrain gradients, and ecosystem-type information associated with spatial FVC heterogeneity. All environmental variables were spatially matched with FVC products and resampled to a unified spatial resolution of 250 m to ensure pixel-level correspondence.

All environmental variables were spatially matched with FVC products and resampled to a unified spatial resolution of 250 m to ensure consistency between predictor variables and vegetation observations. This spatial harmonisation ensured pixel-level correspondence among the response and predictor variables. However, spatial alignment to a common grid does not eliminate differences in the native spatial support of the datasets. Climate variables represent relatively coarse-scale environmental conditions, whereas DEM and land-cover datasets provide comparatively finer-scale spatial information. These differences in native spatial resolution were therefore considered when interpreting the relative contributions derived from the XGBoost–SHAP analysis. Accordingly, the SHAP-derived importance values represent the contribution of each predictor at its available spatial information scale after harmonisation, rather than a direct measure of intrinsic ecological importance that is independent of spatial resolution.

A spatial sampling strategy was subsequently applied to construct the machine-learning dataset. Each sample consisted of one annual growing-season maximum FVC and its corresponding environmental variables. The final dataset was used as input for XGBoost modelling, with FVC as the dependent variable and environmental factors as independent variables. To minimise inconsistencies that might result from different variable types, continuous variables (such as temperature, precipitation, DEM, and slope) were retained as numeric independent variables. Land-cover categories were converted into dummy variables using one-hot encoding before XGBoost modelling, avoiding artificial ordinal relationships among different land-cover classes. Because these categorical variables represent ecosystem structural conditions that are partly related to vegetation status, their SHAP contributions were interpreted as contextual or explanatory information rather than independent causal effects on FVC.

#### 4.6.2. XGBoost Model Development

XGBoost is an optimised gradient boosting decision tree algorithm capable of capturing nonlinear relationships and interactions among ecological variables [31,32]. In this study, XGBoost was employed to evaluate the capability of different environmental variable combinations to represent observed FVC spatial variability. To evaluate the contribution of different environmental variable groups to FVC prediction, three XGBoost models with gradually increasing complexity were constructed:

(1) Climate-only model

The climate-only model considered only climatic variables:

(15) FVC = f (PRE, TMEAN, TMIN, TMAX, PDSI)

This model was used to evaluate the explanatory power of climatic conditions alone for vegetation spatial variation.

(2) Climate–terrain model

The climate–terrain model incorporated topographic variables:

(16) FVC = f (TMEAN, TMIN, TMAX, PRE, PDSI, DEM, Slope, Aspect)

This model was designed to quantify the additional explanatory power provided by topographic heterogeneity.

(3) Full ecological model

The full ecological model integrated climatic, topographic, and land-cover information:

(17) FVC = f (TMEAN, TMIN, TMAX, PRE, PDSI, DEM, Slope, Aspect, LC)

This model represents the comprehensive environmental association framework of spatial FVC variability by considering both environmental gradients and ecosystem structure.

All XGBoost models were implemented in a Python 3.14.7 environment. The objective function was set as squared-error regression. After removing pixels with missing values, the final modelling dataset consisted of 239,736 valid samples. These samples were used for XGBoost training and independent evaluation. Model performance was evaluated using three commonly used statistical metrics: coefficient of determination (R^2^), root mean square error (RMSE), and mean absolute error (MAE).

Coefficient of determination ($R^{2}$):

(18) R2 = 1−∑(yi−yi^)2∑(yi−y¯)2

Root mean square error (RMSE):

(19) RMSE = 1n∑(yi − yi^)2

Mean absolute error (MAE):

(20) MAE = 1n∑yi − yi^

where $y_{i}$ represents observed FVC values, yi^ represents predicted FVC values, $\bar{y}$ represents the mean observed FVC value, and n represents the number of samples. Higher $R^{2}$ values and lower RMSE and MAE values indicate better model predictive performance. Because spatial autocorrelation is an inherent property of environmental and remotely sensed variables, spatial blocking cannot completely eliminate spatial dependence within the study landscape. Therefore, the XGBoost performance metrics were interpreted as an assessment of model generalisation under spatially separated sampling rather than as a complete test of spatial independence.

#### 4.6.3. Hyperparameter Optimisation

To avoid arbitrary parameter selection, XGBoost hyperparameters were optimised using RandomizedSearchCV with spatial cross-validation rather than being fixed a priori. The search was performed with 5-fold spatial GroupKFold cross-validation, in which all samples belonging to the same spatial block were kept together in either the training or the validation fold. The search space included the number of estimators (200–1000), learning rate (0.01–0.2), maximum tree depth (3–10), subsampling ratio (0.6–1.0), column sampling ratio (0.6–1.0), minimum child weight (1–7), and gamma (0–0.5). The optimal parameter combination was selected based on the highest cross-validated R^2^ and then applied consistently to all three models and to the three sub-period analyses. The final optimal hyperparameters are reported in Appendix A Table A3. This procedure ensures that the reported model performance is not dependent on a single arbitrary parameter set and that the hyperparameters are compatible with the spatial structure of the dataset.

#### 4.6.4. Spatial Block Cross-Validation

To account for the spatial structure of the pixel-based dataset, spatially separated sampling units were used for model training and testing. Geographic coordinates were used to assign samples to spatial blocks, and the blocks rather than individual pixels were randomly divided into training and testing subsets. Consequently, spatially adjacent pixels within the same block were not independently allocated to both subsets.

The spatial block size was not set arbitrarily but was determined based on the spatial autocorrelation range of FVC. An exponential semivariogram model was fitted to the FVC data to estimate the distance over which spatial autocorrelation remained substantial. The estimated autocorrelation range was then used to guide block size selection, following the principle that the block size should be at least as large as the autocorrelation range to ensure that training and testing samples are spatially independent. To further evaluate the sensitivity of model performance to block size, a series of candidate block sizes (0.2°, 0.3°, 0.5°, 0.8°, and 1.0°) were tested. For each block size, the number of spatial blocks, R^2^, and RMSE were recorded. Based on this sensitivity analysis, a block size of 0.5° was selected, as it was larger than the estimated autocorrelation range and provided stable model performance across the candidate sizes. The block size sensitivity results are provided in Appendix A Table A4. After selecting the 0.5° block size, spatially grouped samples were divided into approximately 80% training blocks and 20% testing blocks, with all samples within a block assigned to the same subset.

Using the selected block size, spatial blocks were randomly assigned to training (80%) and testing (20%) subsets via GroupShuffleSplit. This strategy was adopted to reduce potential information leakage caused by spatially neighbouring observations and to provide a more conservative assessment of model generalisation. Although spatial dependence cannot be completely excluded, the use of spatially separated testing samples reduces the likelihood that the reported model performance is driven solely by direct neighbourhood overlap between training and testing observations.

#### 4.6.5. SHAP-Based Interpretation of Environmental Associations with FVC

Although machine-learning models provide strong predictive performance, their internal decision-making processes are often difficult to interpret. Therefore, the SHapley Additive exPlanations (SHAP) framework was applied to quantify the contribution of individual environmental variables to XGBoost predictions. SHAP is based on cooperative game theory and decomposes the prediction output into the contribution of each predictor variable. For a given sample, the predicted FVC value can be expressed as [32]:

(21) $$f(x)=\emptyset_{0}+\sum_{i=1}^{m}\emptyset_{i}$$

where f(x) represents the model prediction, $\emptyset_{0}$ represents the baseline prediction, and $\emptyset_{i}$ represents the marginal contribution of the i-th environmental variable.

The overall importance of each environmental factor was quantified using the mean absolute SHAP value:

(22) $${Importance}_{i}=\frac{1}{n}\sum_{j=1}^{n}\left|{SHAP}_{ij}\right|$$

where ${SHAP}_{ij}$ represents the SHAP value of variable i for sample j.

The mean absolute SHAP values were further normalised to calculate the relative importance of each environmental factor:

(23) $${\mathrm{Relative}\;\mathrm{Importance}}_{i}\;=\;\frac{{\mathrm{Importance}}_{i}}{\sum {\mathrm{Importance}}_{i}}\;\times \;100\%$$

A larger mean absolute SHAP value indicates a stronger contribution of the corresponding environmental factor to FVC spatial variation.

SHAP mean absolute values were calculated for each predictor to quantify its relative contribution to the XGBoost model output. Predictors were ranked according to their mean absolute SHAP values, and the complete ranking was visualised to facilitate comparison among climatic, topographic, and land-cover variables. In addition, SHAP dependence plots were used to characterise nonlinear response patterns between dominant environmental factors and FVC. These analyses provided insights into whether specific environmental variables exerted positive, negative, or threshold-dependent effects on FVC.

#### 4.6.6. Period-Specific Consistency Analysis of Environmental Associations with FVC

To examine period-specific differences in the environmental associations represented by the XGBoost models, the study period was divided into three sub-periods: early period (2001–2008), middle period (2009–2016), and recent period (2017–2024). Separate XGBoost models were developed for each period using the same environmental predictor system. Model performance and SHAP-derived variable importance were compared among periods to characterise differences in model attribution. For each period, model accuracy was assessed using R^2^, RMSE, and MAE. The relative contribution of environmental variables was quantified using SHAP analysis.

### 4.7. Statistical Analysis and Software

All remote sensing preprocessing, NDVI compositing, FVC estimation, climate lag analysis, and spatial operations were performed using the Google Earth Engine (GEE) platform (accessed on 29 July 2026). All spatial datasets were processed using the WGS84 coordinate reference system. Spatial resampling, raster alignment, and pixel-based analysis were conducted to ensure consistency among remote sensing, climate, topographic, and land-cover datasets. Machine-learning modelling, statistical analysis, and visualisation were conducted using Python 3.14.7. The major Python packages included NumPy 2.5.2, Pandas 3.0.5, Scikit-learn 1.9.0, XGBoost 3.4.1, SHAP 0.52.0, and Matplotlib 3.11.1.

## 5. Conclusions

This study integrated remote sensing and climate datasets with statistical trend analysis, lagged correlation analysis, and interpretable machine learning to characterise FVC dynamics and environmental associations on the Western Sichuan Plateau during 2001–2024. The main conclusions are as follows:

(1) Growing-season FVC exhibited a significant increasing trend during 2001–2024, although the magnitude and direction of change varied substantially across the study area. The spatial pattern of FVC was characterised by higher coverage in the eastern and southeastern regions and lower coverage in western and high-elevation areas.
(2) Temperature and precipitation showed widespread lagged associations with FVC. The dominance of longer lag periods indicates that antecedent climate anomalies were statistically associated with subsequent vegetation conditions.
(3) The XGBoost–SHAP analysis showed that topographic, land-cover, and climatic variables provided complementary explanatory information for the spatial variability of FVC. DEM, grassland land cover, and mean temperature were among the most influential predictors of the modelled spatial FVC patterns.
(4) Period-specific XGBoost models showed broadly comparable predictive performance, while the relative SHAP contributions of individual variables varied among periods.
(5) Overall, the combined analysis distinguishes long-term temporal trends, lagged climate–vegetation associations, and spatial environmental heterogeneity, providing a more cautious framework for understanding FVC variation across the Western Sichuan Plateau.

## Figures and Tables

**Table 1:** Area statistics of different FVC levels in the Western Sichuan Plateau during 2001–2024.

FVC Level	Proportion (%)	Area (km^2^)
low	7.5909	29,073.74008
relatively low	5.2387	20,064.43617
moderate	11.3830	43,597.78395
relatively high	28.8848	110,630.9106
high	46.9025	179,639.9247

**Table 2:** Area statistics of different FVC change trends during 2001–2024.

FVC Change Trend	Proportion (%)	Area (km^2^)
significant degradation	1.9783%	6494.4375 km^2^
non-significant degradation	21.5026%	70,590.3125 km^2^
stability	12.8971%	42,339.4375 km^2^
non-significant improvement	47.0582%	15,4486.0000 km^2^
significant improvement	16.5638%	54,376.6250 km^2^

**Table 3:** Area statistics of correlation strength between temperature or precipitation and FVC.

Climate Factor	Correlation Strength	Maximum Absolute Correlation Coefficient	Area (km^2^)	Percentage (%)
Temperature	Very weak	0–0.2	7671.8746	2.0031
	Weak	0.2–0.4	170,038.1323	44.3956
	Moderate	0.4–0.6	191,116.6760	49.8990
	Strong	0.6–0.8	14,151.0984	3.6947
	Very strong	0.8–1	29.0142	0.0076
Precipitation	Very weak	0–0.2	7301.7794	1.9064
	Weak	0.2–0.4	168,855.2747	44.0868
	Moderate	0.4–0.6	193,074.5849	50.4102
	Strong	0.6–0.8	13,741.5381	3.5878
	Very strong	0.8–1	33.6184	0.0088

**Table 4:** Area proportion of optimal lag time between climatic factors and FVC.

Climate Factor	Lag Category	Lag Months	Area (km^2^)	Percentage (%)
Temperature	Immediate	0	39,549.4658	10.3260
	Short-term	1–3	94,616.5466	24.7036
	Medium-term	4–6	73,959.7681	19.3103
	Long-term	7–12	174,881.0150	45.6600
Precipitation	Immediate	0	37,123.8221	9.6927
	Short-term	1–3	83,400.5574	21.7752
	Medium-term	4–6	89,835.3464	23.4553
	Long-term	7–12	172,647.0695	45.0768

**Table 5:** Performance of XGBoost models based on spatially separated testing samples.

XGBoost Model	*R* ^2^	RMSE	MAE
Climate-only	0.2181	0.2650	0.2060
Climate–terrain	0.5716	0.1961	0.1451
Full ecological	0.6393	0.1800	0.1263

Note: R^2^, RMSE, and MAE were calculated using independent testing samples obtained through spatial block-based data partitioning.

**Table 6:** Performance of XGBoost models during different periods.

Period	*R* ^2^	RMSE	MAE
2001–2008	0.6515	0.1783	0.1268
2009–2016	0.6424	0.1789	0.1258
2017–2024	0.6490	0.1764	0.1225

## Data Availability

The datasets used in this study were obtained from publicly available remote-sensing and environmental data sources, including MODIS vegetation products, ERA5-Land climate data, digital elevation data, and MODIS land-cover products. The original datasets can be accessed from their respective data providers. The processed FVC dataset used in this study, as well as the datasets generated for direct use in modelling, are available at: https://zenodo.org/records/22170070 (accessed on 23 September 2026).

## Appendix A

**Table A1:** Summary of datasets used in this study.

Dataset	Abbreviation	Source	Spatial Resolution	Temporal Resolution	Period	Purpose
Normalised Difference Vegetation Index	NDVI	https://developers.google.com/earth-engine/datasets/catalog/MODIS_061_MOD13Q1 (accessed on 29 July 2026)	250 m	16-day	2001–2024	NDVI extraction and FVC estimation
Fractional Vegetation Cover	FVC	Calculated from NDVI	250 m	Annual	2001–2024	Vegetation dynamics assessment
ERA5-Land	TMEAN, TMIN, TMAX, PRE	https://developers.google.com/earth-engine/datasets/catalog/ECMWF_ERA5_LAND_MONTHLY_AGGR (accessed on 29 July 2026)	~11,132 m	Monthly	2001–2024	Temperature and precipitation analysis
Drought Index	PDSI	https://developers.google.com/earth-engine/datasets/catalog/IDAHO_EPSCOR_TERRACLIMATE (accessed on 29 July 2026)	~4638.3 m	Monthly	2001–2024	Drought stress characterisation
Digital Elevation Model	DEM	https://developers.google.com/earth-engine/datasets/catalog/USGS_SRTMGL1_003 (accessed on 29 July 2026)	30 m	Static	—	Topographic effects
Topographic Factors	Slope, Aspect	Derived from DEM	30 m	Static	—	Terrain heterogeneity analysis
Land Cover	LC	https://developers.google.com/earth-engine/datasets/catalog/MODIS_061_MCD12Q1 (accessed on 29 July 2026)	500 m	Annual	2001–2024	Land-cover classification
Study boundary	ROI	Western Sichuan Plateau boundary	Vector	Static	—	Spatial analysis

**Table A2:** IGBP classification criteria and the meaning of the codes used in this study.

Value	Code	Description
1	LC_1	Evergreen Needleleaf Forests: dominated by evergreen conifer trees (canopy >2 m). Tree cover > 60%.
2	LC_2	Evergreen Broadleaf Forests: dominated by evergreen broadleaf and palmate trees (canopy >2 m). Tree cover > 60%.
3	LC_3	Deciduous Needleleaf Forests: dominated by deciduous needleleaf (larch) trees (canopy >2 m). Tree cover > 60%.
4	LC_4	Deciduous Broadleaf Forests: dominated by deciduous broadleaf trees (canopy >2 m). Tree cover > 60%.
5	LC_5	Mixed Forests: dominated by neither deciduous nor evergreen (40–60% of each) tree type (canopy >2 m). Tree cover > 60%.
6	LC_6	Closed Shrublands: dominated by woody perennials (1–2 m height) >60% cover.
7	LC_7	Open Shrublands: dominated by woody perennials (1–2 m height) 10–60% cover.
8	LC_8	Woody Savannas: tree cover 30–60% (canopy >2 m).
9	LC_9	Savannas: tree cover 10–30% (canopy >2 m).
10	LC_10	Grasslands: dominated by herbaceous annuals (<2 m).
11	LC_11	Permanent Wetlands: permanently inundated lands with 30–60% water cover and >10% vegetated cover.
12	LC_12	Cropland.
13	LC_13	Urban and Built-up Lands: at least 30% impervious surface area including building materials, asphalt and vehicles.
14	LC_14	Cropland/Natural Vegetation Mosaics: mosaics of small-scale cultivation 40–60% with natural tree, shrub, or herbaceous vegetation.
15	LC_15	Permanent Snow and Ice: at least 60% of area is covered by snow and ice for at least 10 months of the year.
16	LC_16	(sand, rock, soil) areas with less than 10% vegetation.
17	LC_17	Water Bodies: at least 60% of area is covered by permanent water bodies.

**Table A3:** Optimal XGBoost hyperparameters.

Parameter	Optimal Value
n_estimators	1000
learning_rate	0.03
max_depth	6
subsample	0.6
colsample_bytree	0.7
min_child_weight	7
gamma	0.3

**Table A4:** Block size sensitivity analysis.

Block Size (°)	Number of Blocks	R^2^	RMSE
0.2	737	0.2497	0.2338
0.3	352	0.1881	0.2611
0.5	140	0.2188	0.2649
0.8	65	0.3788	0.2248
1.0	44	0.2175	0.2603
